# Improving ecosystem health in highly altered river basins: a generalized framework and its application to the Mississippi-Atchafalaya River Basin

**DOI:** 10.3389/fenvs.2024.1332934

**Published:** 2024-02-22

**Authors:** Eileen L. McLellan, Kelly M. Suttles, Kristen L. Bouska, Jamelle H. Ellis, Joseph E. Flotemersch, Madison Goff, Heather E. Golden, Ryan A. Hill, Tara R. Hohman, Shamitha Keerthi, Richard F. Keim, Barbara A. Kleiss, Tyler J. Lark, Bryan P. Piazza, Alisha A. Renfro, Dale M. Robertson, Keith E. Schilling, Travis S. Schmidt, Ian R. Waite

**Affiliations:** 1 Environmental Defense Fund, Washington, DC, United States; 2 Environmental Defense Fund, Raleigh, NC, United States; 3 U.S. Geological Survey, Upper Midwest Environmental Sciences Center, La Crosse, WI, United States; 4 Theodore Roosevelt Conservation Partnership, Washington, DC, United States; 5 U.S. Environmental Protection Agency, Office of Research and Development, Cincinnati, OH, United States; 6 School for Environment and Sustainability, University of Michigan, Ann Arbor, MI, United States; 7 U.S. Environmental Protection Agency, Office of Research and Development, Corvallis, OR, United States; 8 Audubon Upper Mississippi River, Audubon Center at Riverlands, West Alton, MO, United States; 9 The Nature Conservancy, Arlington, VA, United States; 10 School of Renewable Natural Resources, Louisiana State University, Baton Rouge, LA, United States; 11 Department of River Coastal Science and Engineering, Tulane University, New Orleans, LA, United States; 12 Center for Sustainability and the Global Environment, University of Wisconsin, Madison, WI, United States; 13 The Nature Conservancy, Baton Rouge, LA, United States; 14 National Wildlife Federation, New Orleans, LA, United States; 15 U.S. Geological Survey, Upper Midwest Water Science Center, Madison, WI, United States; 16 IIHR-Hydroscience and Engineering, University of Iowa, Iowa City, IA, United States; 17 U.S. Geological Survey, Wyoming-Montana Water Science Center, Helena, MT, United States; 18 U.S. Geological Survey, Oregon Water Science Center, Portland, OR, United States

**Keywords:** resilience, river health, ecosystem health, river basin, landscape-level indicators, adaptive management

## Abstract

Continued large-scale public investment in declining ecosystems depends on demonstrations of “success”. While the public conception of “success” often focuses on restoration to a pre-disturbance condition, the scientific community is more likely to measure success in terms of improved ecosystem health. Using a combination of literature review, workshops and expert solicitation we propose a generalized framework to improve ecosystem health in highly altered river basins by reducing ecosystem stressors, enhancing ecosystem processes and increasing ecosystem resilience. We illustrate the use of this framework in the Mississippi-Atchafalaya River Basin (MARB) of the central United States (U.S.), by (i) identifying key stressors related to human activities, and (ii) creating a conceptual ecosystem model relating those stressors to effects on ecosystem structure and processes. As a result of our analysis, we identify a set of landscape-level indicators of ecosystem health, emphasizing leading indicators of stressor removal (e.g., reduced anthropogenic nutrient inputs), increased ecosystem function (e.g., increased water storage in the landscape) and increased resilience (e.g., changes in the percentage of perennial vegetative cover). We suggest that by including these indicators, along with lagging indicators such as direct measurements of water quality, stakeholders will be better able to assess the effectiveness of management actions. For example, if both leading and lagging indicators show improvement over time, then management actions are on track to attain desired ecosystem condition. If, however, leading indicators are not improving or even declining, then fundamental challenges to ecosystem health remain to be addressed and failure to address these will ultimately lead to declines in lagging indicators such as water quality. Although our model and indicators are specific to the MARB, we believe that the generalized framework and the process of model and indicator development will be valuable in an array of altered river basins.

## 1 Introduction

Public concern over ecosystem decline has prompted investments to improve the health of select ecosystems. In the United States (U.S.), Federal and state governments spend billions of dollars per year to benefit the Chesapeake Bay, Florida Everglades, Columbia River and the Louisiana coast. In Europe, the European Commission has passed ambitious legislation to restore natural habitats across 20% of land and sea by 2030 (European Commission, 2023), and Australia has committed to restoring its largest river basin, the Murray-Darling (SBS News, 2023), as well as the Great Barrier Reef (Readfearn, 2022). Support for continued public investment in such projects will require some indication of “success.” Success in these efforts has often been narrowly expressed in terms of restoring ecosystem structure—delineated in terms such as hectares of specific habitat, populations of iconic species, measures of biodiversity, or water quality targets—to historic (e.g., “pre-settlement”) conditions.

The 1972 Clean Water Act in the U.S., which seeks to “restore and maintain the chemical, physical and biological integrity of the Nation’s waters” is an example of an early attempt to promote restoration of historic ecosystem conditions. Across multiple levels of government, an elaborate set of water quality monitoring programs, variously focused on water chemistry, aquatic habitat, and biological integrity has been developed to track “success”. Also in response to the Clean Water Act a multi-billion dollar industry has developed to promote stream, wetland and river restoration—but with its emphasis on restoring geomorphic “form” it has not, in general, led to improved water quality or recolonization of “restored” streams and rivers by desired biota (Bernhardt and Palmer, 2011). This is perhaps not surprising: in many ecosystems, human demands for food, water, energy and housing have dramatically altered fundamental ecosystem processes, making it unlikely that historic conditions can ever be restored and sustained (Brown et al., 2018; Best, 2019). Climate change and its multi-faceted impacts further complicate restoration efforts (Tonkin et al., 2019). Together, these constraints challenge the idea that success—defined in terms of restoring the past—can now be achieved. In this paper, we propose a broader definition of success for ecosystem management that is grounded in current definitions of ecosystem health.

Our definition follows from the shift in emphasis in restoration ecology towards “process-based” restoration in which the goal is not re-creation of historic conditions, but rather restoration and/or enhancement of ecological processes that support biodiversity (Beechie et al., 2010; Palmer et al., 2014; Palmer and Ruhi, 2019). Process-based restoration recognizes that ecosystems are dynamic, that restoration methods must be adaptive and that future outcomes cannot be guaranteed. To avoid confusion with efforts to restore historic conditions, such efforts are sometimes termed “ecosystem renovation” (Prober et al., 2019) or “ecosystem design” (Higgs, 2017), or even “rewilding” (du Toit and Pettorelli, 2019; Perino et al., 2019; Rideout et al., 2021). In rewilding, the emphasis is on restoring ecological processes in a self-organizing system such that minimal intervention is needed to sustain those processes (du Toit and Pettorelli, 2019).

In parallel with scientists’ increasing emphasis on ecosystem processes has come greater societal interest in ecosystem function, and the goods and services that ecosystems provide to humans (Garland et al., 2021). In addition, growing awareness of climate change and its effects has led to increased interest in resilience, i.e., the ability of a system to tolerate and recover from disturbance (Grantham et al., 2019). The growing focus on ecological processes, ecosystem services and resilience has been integrateds under the broad umbrella of “ecosystem health” (Flotemersch et al., 2016; Kuehne et al., 2017; Clapcott et al., 2020; Verdonschot et al., 2020).

For example, Flotemersch et al. (2016) defined the ecological integrity of watersheds as “the capacity of a watershed to support and maintain the full range of ecological processes and functions essential to the sustainability of both biodiversity and the watershed resources and services provided to society.” Likewise, New Zealand’s National Policy Statement on Freshwater Management defines healthy freshwater ecosystems as those where “ecological processes are maintained, there is a range and diversity of native flora and fauna, and there is resilience to change” (Clapcott et al., 2020). Implicit in both definitions is the need to address the drivers of ecosystem degradation to maintain health; in large river basins, such drivers include large-scale land use changes (Sundermann et al., 2013) and river engineering (Sofi et al., 2020).

We combine elements of these definitions and specify ecosystem health as: “*the capacity of a system to sustain the full range of ecosystem processes and functions needed to support biodiversity and ecosystem services and to ensure that these processes are resilient to change*.” Given our emphasis on ecological processes and functions, our perspective on biodiversity is on the diversity of functional traits across a species assemblage, not the presence or abundance of individual or even native species (though we recognize that some agencies may have legislative mandates to manage for specific species).

Following Clapcott et al. (2020) and Flotemersch et al. (2016) we overtly include resilience as a component of ecosystem health. Resilience is integral to ecosystem health because it is fundamentally linked to the continuance of ecosystem processes and functions (Holling, 1973; Oliver et al., 2015). Assuming that an ecosystem is in a desirable state (perhaps recognized as desirable by the provision of specific ecosystem services), a healthy ecosystem will be able to respond to short- and long-term disturbances without shifting into a less-desirable state in which it is unable to support the desired ecosystem processes and services. For example, a wetland which supports nutrient cycling may respond to a disturbance by a change in the type of wetland vegetation, but so long as the post-disturbance wetland is still able to provide nutrient cycling we would consider it resilient. We thus define “resilience” as the ability to maintain ecosystem processes in the face of disturbances (stressors) through changes in ecosystem structure; ecosystems may “bounce forward” through adaptation or transformation, rather than retaining or returning to their pre-disturbance structure (“bouncing back”). This definition of resilience draws from the work of Oliver et al. (2015) and Lane et al. (2023) and is aligned with ecological rather than engineering resilience (Angeler and Allen, 2016). We focus here on general resilience: the “resilience of any and all parts of a … system to all kinds of shocks, including novel ones” (Folke et al., 2010), not specified resilience (“the resilience of what [specific aspects of ecosystem condition] to what [specific stressors]?” Folke et al., 2010).

Recent papers have explored the applications and implications of resilience theory to river systems. Historically, large river basins have been managed as static systems, rigidly engineered by way of concrete infrastructure and operated to produce a single desired outcome such as hydropower or flows for navigation. In contrast, a resilience perspective sees rivers as dynamic systems, able to adapt and change in response to changes in external stressors such as climate change—but at risk of changing to less-desirable states once that adaptive capacity is exceeded (Grantham et al., 2019). Consequently, there is growing interest in more flexible and adaptive approaches to river management that better support and sustain ecosystem processes and services (Poff et al., 2016).

**FIGURE 1:**
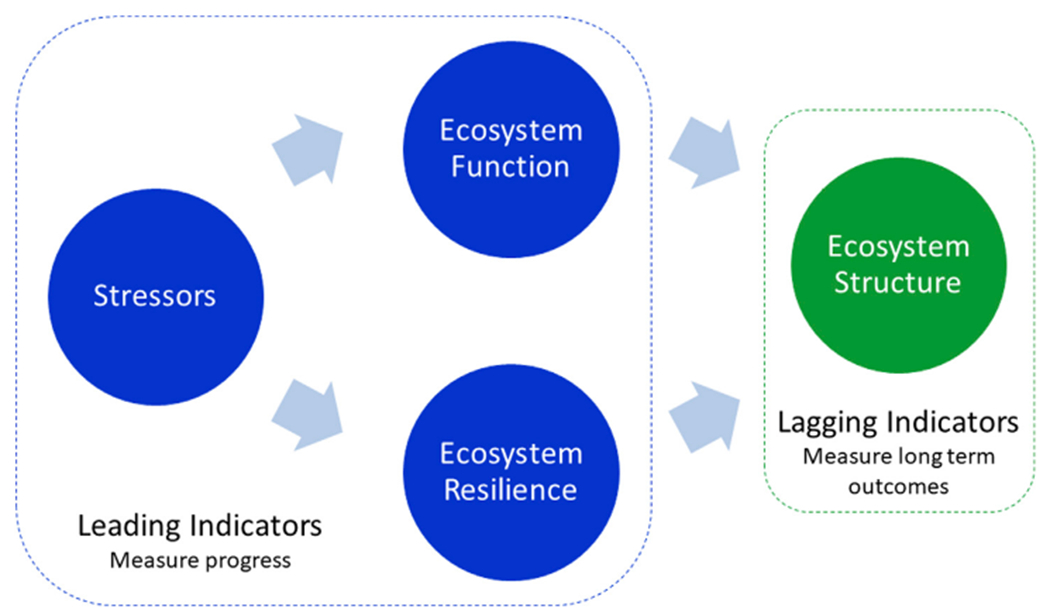
Relationship between leading and lagging indicators of ecosystem health. We posit that changes in leading indicators, which measure changes in fundamental elements of ecosystem health, serve as an early warning system for likely changes in ecosystem structure.

Our goal in this paper is to show how this definition of ecosystem health can be used to guide the management of large river basins. First, we present a management framework for improving ecosystem health based on reducing the drivers of ecosystem degradation, enhancing ecosystem function (processes), and increasing resilience. Second, we use this framework to develop a conceptual ecosystem model for a highly altered, large river basin: the Mississippi-Atchafalaya River Basin (MARB) in the U.S. Third, we show how the framework and conceptual ecosystem model can be used to identify landscape-level indicators of ecosystem health. In developing indicators, we focus on “leading” indicators (*sensu* Hall et al., 2014; 2019; Ota et al., 2021) that drive changes in ecosystem structure, in contrast to “lagging” indicators which measure how ecosystem structure changes in response to these drivers (Figure 1). We anticipate these leading indicators will be of interest to managers as they are both forward-looking (i.e., predict the likely future direction of change in ecosystem condition) and directly related to management actions. Finally, we explore how “leading” indicators could be integrated into existing monitoring programs to create an adaptive management system that can be used to test the effects of management actions and inform any needed changes.

## 2 A generalized framework for improving ecosystem health

**FIGURE 2:**
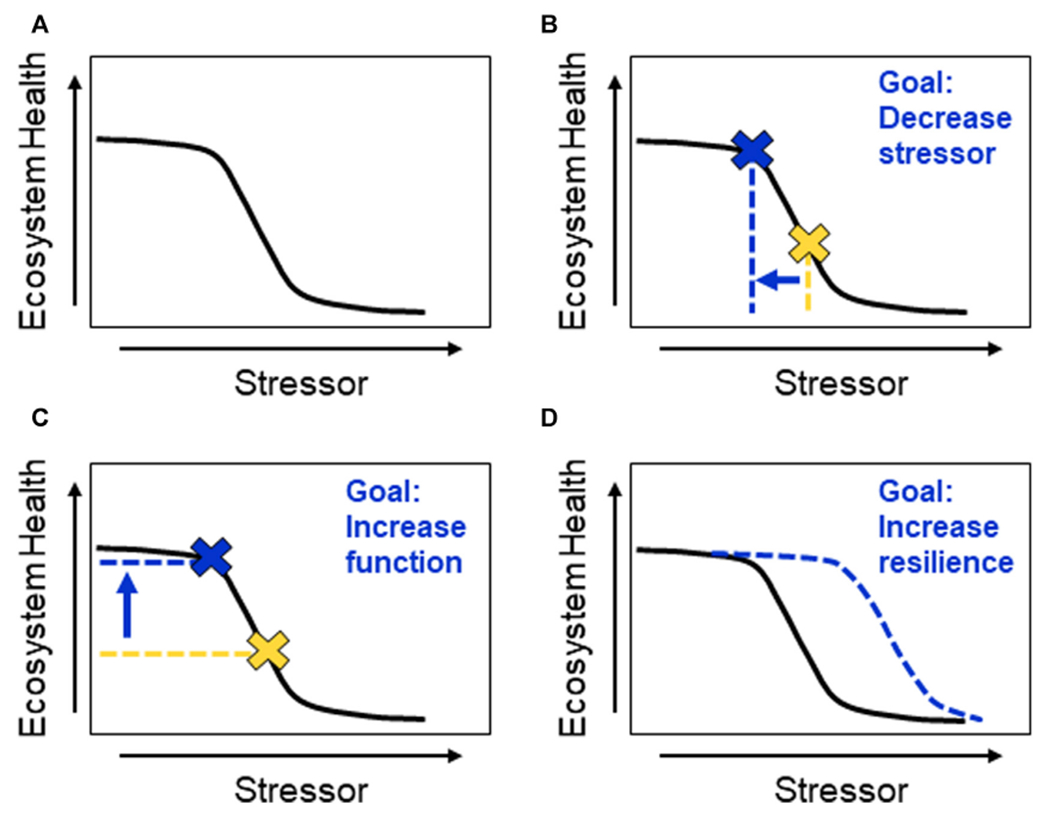
Stressor-response curves illustrating the functional equivalence of changes in ecosystem stressors, function and resilience **(A)**. A simplistic representation of the relationship between stressors and ecosystem function, adapted from the stressor-response curves of Larned and Schallenberg (2019), showing that increased stressors degrade ecosystem health. In the following panels, the dotted yellow lines and associated yellow cross represent levels of stressors and ecosystem health prior to management interventions, while the dotted blue lines and associated blue cross represent the same levels after management interventions **(B)**. Management actions to reduce stressors lead to an increase in ecosystem health **(C)**. Management actions to improve ecosystem function also improve ecosystem health and are functionally equivalent to reductions in stressors **(D)**. Management actions that enhance resilience increase the ecosystem’s ability to absorb stress, effectively shifting the stressor-response curve to the right (dashed blue curve); this is equivalent to increasing ecosystem function at current stressor levels or maintaining current levels of ecosystem function at higher stressor levels.

We ground our discussion of ecosystem (here, river basin) health in the context of watershed (river basin) resilience (Lane et al., 2023). Specifically, we assume there are multiple stable watershed states (“basins of attraction” in Lane et al., 2023) for a given system, each with its own structural and functional characteristics. River basin resilience, as we define it, refers to the ability to maintain ecosystem processes (functions) in the face of disturbances (stressors) through changes in ecosystem structure. In contrast, “regime change” refers to a transition from one stable state to another with different structures and functions in response to disturbances. In general, increased resilience is viewed positively and regime change negatively. However, we recognize that there are situations in which the state of the current system is considered undesirable, therefore, regime change is desired and increased resilience is problematic (Du and Pettorelli, 2019). For example, Markolf et al. (2018) describe how in many river basins, including the MARB, historic emphasis on flood risk reduction through engineered infrastructure has created “lock-in” to an engineering approach that may be maladaptive in the face of climate change. Likewise, Oliver et al. (2018) describe how lock-in and path dependency reinforce unhelpful resilience in food systems. We focus, here, on avoidance of unwanted regime change and the benefits of increased resilience in preventing unwanted change. Achieving this goal requires a reduction in disturbances (stressors) and/or increases in river basin function and/or resilience, which we illustrate in Figure 2.

Figure 2A shows a hypothetical stressor-response curve for an ecosystem, where the response is some measure of ecosystem health, based on the work of Larned and Schallenberg (2019). The shapes of stressor-response curves can be quite variable (linear, quadratic, threshold, etc.), depending on the specific stressor and response, and may even vary regionally for a given set of stressors and responses (Larned and Schallenberg, 2019), but they are often nonlinear (Selkoe et al., 2015). Crucially, the effect on ecosystem health depends only on the inverse relationship between ecosystem health and stressor rather than the exact shape of the curve, i.e., an increase in a stressor leads to a degradation of ecosystem health. The remaining panels of Figure 2 schematically illustrate the effect of management actions to reduce stressors (Figure 2B), improve ecosystem function (Figure 2C) and increase resilience (Figure 2D).

Management actions aimed at reducing stressors improve ecosystem health (Figure 2B), as do activities that presumptively increase ecosystem function, such as wetland restoration n (Figure 2C).Note that ecosystem responses are identical in Figures 2B,C, meaning that managers can choose to focus on stressor reduction or increased function and achieve the same benefits for ecosystem health. Management actions that improve the attributes of resilience increase the system’s ability to absorb stress, effectively shifting the stressor-response curve to the right (dotted blue line in Figure 2D). This is equivalent to increasing ecosystem health at current stressor levels (by increasing the latitude and/or resistance of the basin of attraction (*sensu* (Lane et al., 2023) or to maintaining current levels of ecosystem health at higher stressor levels.

**FIGURE 3:**
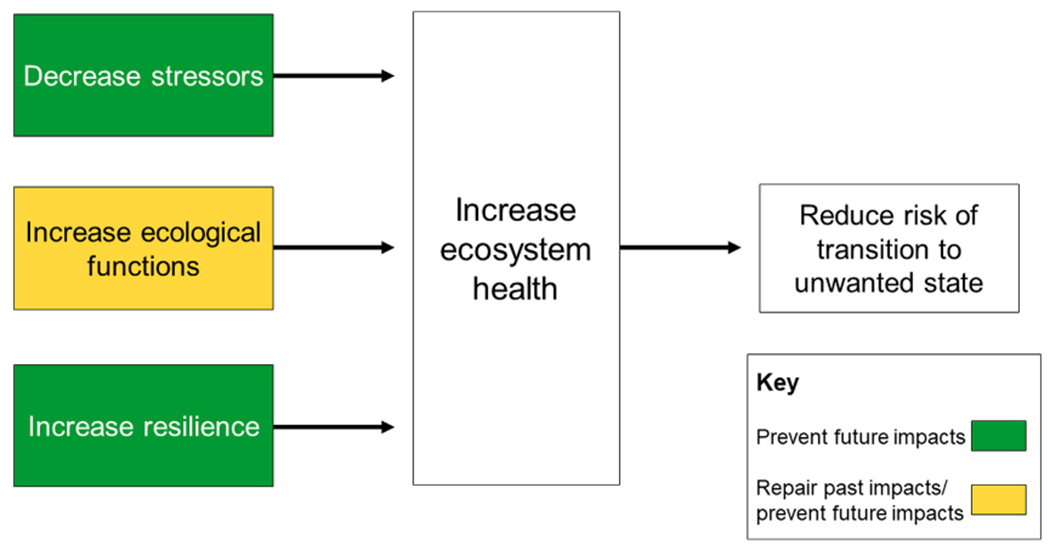
A generalized framework for improving ecosystem health.

Given that most ecosystems have both experienced historic degradation and will continue to be stressed, there is value in combining these different categories of management interventions (Figure 3). Restoring and enhancing ecosystem functions can help to address effects (reductions of function) from past disturbances, while all three types of management action can limit future impacts. All three categories of management action are needed to improve and maintain ecosystem health and reduce the risk of transition to an unwanted state. For any given ecosystem, the task is to translate this generalized framework into specific actions based on specific ecosystem stressors, ecological functions and attributes of resilience and then work with stakeholders to identify those that are the most ecologically meaningful, economically viable and socially acceptable.

## 3 The Mississippi-Atchafalaya River Basin

**FIGURE 4:**
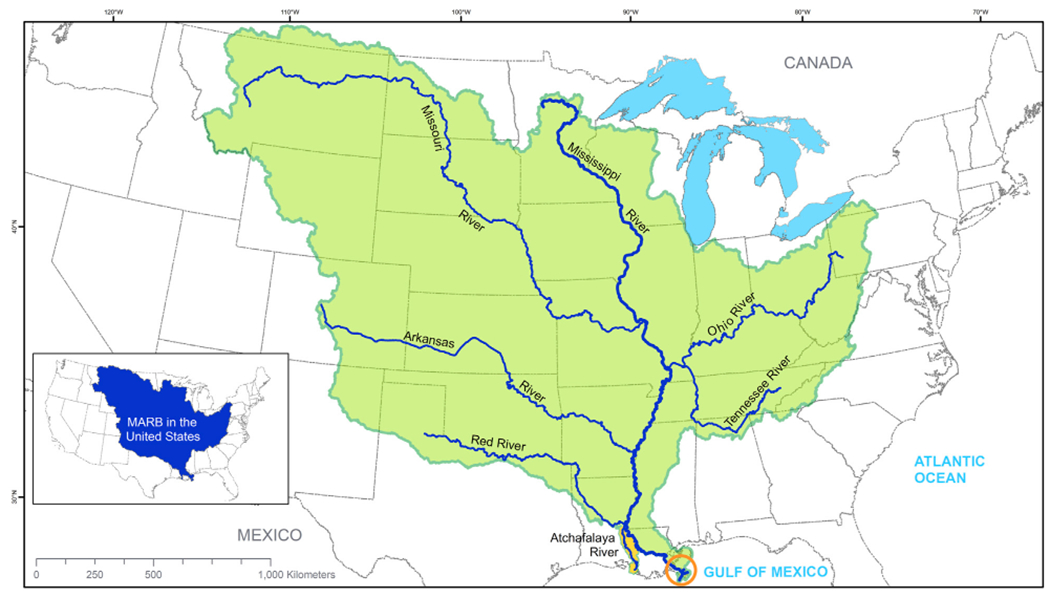
The Mississippi-Atchafalaya River Basin as a hydrologically defined system. The mainstem of the Mississippi River and its major tributaries are shown in blue, and the associated drainage area in green. The junction of the Mississippi and Ohio Rivers separates the Upper and Lower Mississippi River Basin. The Atchafalaya River subwatershed, shown in gold, occupies a historic channel of the Mississippi River. The Mississippi River Delta is circled in orange.

Measuring 3.2 million km^2^, the MARB (Figure 4) is the largest watershed in the U.S. and fourth largest in the world. Significant differences in topography, native vegetation, land use and river geomorphology separate the Upper Mississippi River Basin, above the confluence of the Ohio and Mississippi Rivers, and the Lower Mississippi River Basin. The lowermost portion of the Lower Mississippi River Basin, the Mississippi River Delta, is an estuarine system connecting the MARB to the northern Gulf of Mexico. The Atchafalaya River Basin in south-central Louisiana, occupying the floodplain of a historic distributary channel of the Mississippi River, is the nation’s largest river swamp.

**FIGURE 5:**
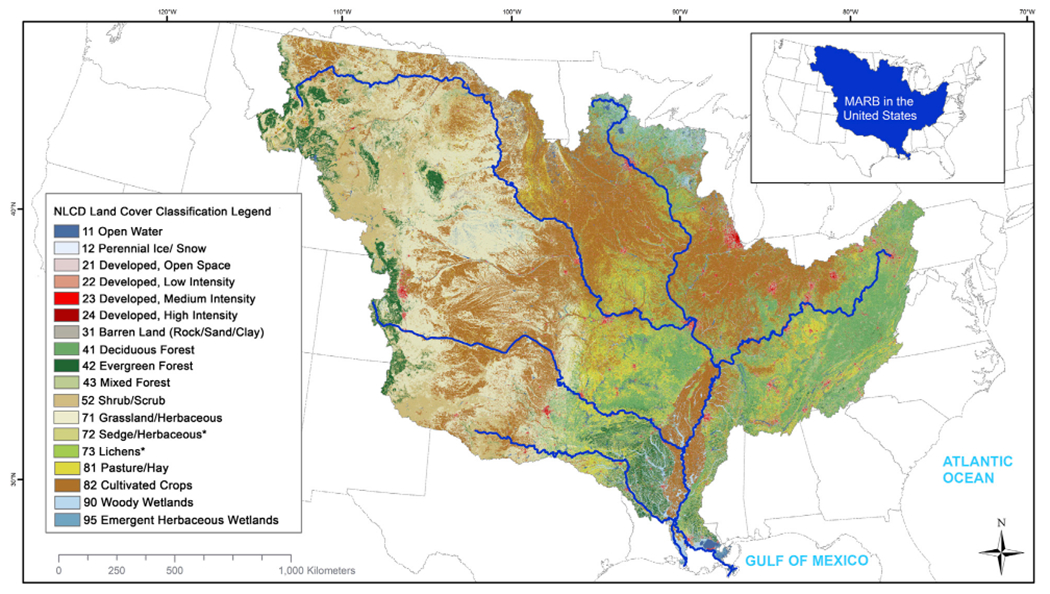
Land use in the Mississippi-Atchafalaya River Basin with 2019 National Land Cover Database (NLCD) classifications.

The MARB is a hydrologic system, in which water, nutrients and sediment from hydrologically connected patches in the uplands move downstream through wetlands and streams to larger tributary rivers, the mainstem Mississippi River, the Atchafalaya River and finally the Delta and Gulf of Mexico (Figure 5). Recent scientific advances help us understand the critical role played by this connected hydrologic system in transporting (and sometimes retaining) water, nutrients and sediment across and within the broader landscape (Leibowitz et al., 2018). We can also view the MARB as an ecological macrosystem (McCluney et al., 2014), consisting of networks of interconnected riverine and upland habitat patches across and between which water, energy, nutrients, sediment, genes and organisms flow. These habitat patches include a variety of types of wetlands, grasslands and other areas of herbaceous vegetation, forests, cultivated cropland, hay and pasture, and developed land (Figure 5). It is critical to understand the important linkages between the MARB’s upland ecological and networked hydrological (aquatic) systems, because riverine health is strongly influenced by upland conditions (Allan, 2004).

The ecological health of the MARB has changed over the past 300 years in response to a variety of natural and (especially) anthropogenic drivers (DuBowy, 2013; Yin et al., 2023). Large-scale transformation of the Upper Basin has been driven by agricultural intensification, primarily the production of row crops (predominantly corn and soybeans), with more recent growth in intensive animal production (Jones et al., 2019) as well as the construction within the river corridor of dams and associated locks to provide a reliable commercial navigation system. Within the Lower Mississippi River Basin, the former river floodplain—the Mississippi Alluvial Valley—has been transformed by large-scale levee creation for river transportation and flood control (Munoz et al., 2018), and by the replacement of almost 8.1 million hectares of floodplain forest by agricultural production, the latter supported by large-scale groundwater withdrawals (Yasarer et al., 2020). In the lowermost MARB, the Delta region has been transformed by a combination of coastal erosion, land subsidence, global sea-level rise, oil and gas extraction, and re-engineering of river channels (Xu et al., 2018).

At present, governance of the MARB is highly fragmented across numerous Federal and State agencies (Chase, 2011), each with its own area of interest (e.g., water quality, river navigation, flood control). As is well-documented in the literature, management of competing objectives is extremely challenging in river basins that lack a coordinated management structure (Pahl-Wostl et al., 2020). In contrast, river basins with either a unitary management authority (e.g., the Tennessee Valley Authority, the world’s first river basin management authority) or a coordinated, multilevel governance structure (such as examples in the Chesapeake Bay and Lake Champlain) have proven more successful in balancing competing objectives (Kauffman, 2015; Margerum and Robinson, 2015; Baumgardner, 2019; Moore, 2021). Those river basins with a well-developed governance structure have also been successful in accessing Federal funding to achieve management objectives.

Recognizing the need for and value of basin-wide governance, a number of entities have recently proposed creating such a system for the MARB (Reed et al., 2020; McCollum, 2021; Brewer, 2023). Although the proposed pathways to, and detailed operations of, proposed governance structures vary, the authors of this paper anticipate that a basin-wide governance structure for the MARB will be in place in a few years. Further recognizing that any large-scale Federal investment in the MARB will require measurement of success in restoring ecosystem health, our emphasis in this paper is on developing indicators that can be used to assess changes in ecosystem health in the MARB.

## 4 A conceptual ecosystem model for the Mississippi-Atchafalaya River Basin

The value of conceptual ecosystem models for guiding large-scale ecosystem management projects has been well documented in the Comprehensive Ecosystem Restoration Program in south Florida’s Everglades (Ogden et al., 2005). The conceptual model for that ecosystem represented the consensus understanding of the key ecosystem stressors and the ecological responses to those stressors and was used as a key planning tool in designing the restoration effort. In south Florida and elsewhere, conceptual ecosystem models represent a set of “working hypotheses” that can be used to identify priority management actions, research gaps and indicators of ecological change (Thom et al., 2003; Twilley et al., 2008; DiGennaro et al., 2012; Testa et al., 2017).

The essence of our conceptual model for the MARB was to link the seemingly disparate parts of the system—uplands, headwater streams, large rivers, wetlands and all the associated landscape features and habitats—in terms of the flow of water, nutrients, sediment and energy. We sought to build connections between the region’s landscapes and riverscapes (Fausch et al., 2002; Allan, 2004); recognizing that while many of the authors of this paper study only a small portion of the system, these portions are inextricably interconnected to one another and are affected by natural and anthropogenic disruptions at landscape to regional (and even global) scale.

Building on the generalized framework for improving ecosystem health (Figure 3), we used a process of expert elicitation to identify key stressors, ecosystem processes, ecosystem functions and attributes of river basin resilience in the MARB and incorporate these into a conceptual ecosystem model. Briefly, our process consisted of: 1) a set of workshops in August—December 2021 that brought scientists from Federal and State agencies, academic institutions, and nonprofit organizations across the MARB together with watershed managers from interstate and regional institutions; and 2) refinement of the model and indicators derived from the model with sub-groups of workshop participants.

### 4.1 Ecosystem components and structure in the Mississippi-Atchafalaya River Basin

We considered the following components that play key roles in supporting ecological processes and functions in the MARB:

- prairies, grasslands and forests in the uplands;
- stream and river channels at all scales from headwater streams to the Delta;
- geographically isolated (non-floodplain) wetlands in the uplands;
- riparian wetlands at the interface between uplands and low-order streams;
- floodplain wetlands at all scales (including bottomland hardwoods); and
- deltaic wetlands at the interface between the Lower Mississippi River and the northern Gulf of Mexico.

The ecosystem structure of the MARB is represented by the diversity of these components and their spatial relationships to one another in networks along which energy, water, sediment, nutrients and organisms can flow. The specifics of ecosystem structure vary tremendously across different regions and scales within the MARB.

### 4.2 Ecosystem stressors in the Mississippi-Atchafalaya River Basin

We identified three broad categories of anthropogenic change relevant to the MARB: land use change, river engineering and climate change (Table 1 and Supplementary Table S1); while we list them separately, we recognize that there are often interactions between them, for example, increasing urbanization can lead to calls for reduced flood risk through river engineering. While both land use change and river engineering occur within the MARB and are potentially amenable to being addressed by actions within the basin, climate change is a global issue that can only be partially addressed through actions within the MARB. However, climate change interacts and intensifies the effects of both land use change and river engineering, so we include it here.

The effects on ecosystems in the MARB often result from interactions between multiple drivers. For example, the conversion of native perennial vegetation to agricultural land (change in land use), facilitated by the installation of artificial drainage and coupled with intensification of agricultural production (e.g., addition of synthetic fertilizers), led to increased nutrient exports from croplands in the Upper Mississippi River Basin (McLellan et al., 2015). Historically, these nutrients might have been deposited or transformed in wetland complexes within the Upper Mississippi River Basin or retained within floodplains on the mainstem Mississippi and smaller rivers. However, drainage of wetlands and hydrologic engineering of river corridors has reduced the nutrient storage and processing functions of the system (Mitsch et al., 2001), thereby increasing nutrient delivery to the mouth of the Mississippi River, which has led to the subsequent formation of a hypoxic zone in the northern Gulf of Mexico (Mitsch et al., 2005). Climate change, through intensification of the hydrologic cycle, is anticipated to make this problem worse (Lu et al., 2020). Likewise, wetland loss in the Mississippi River Delta has resulted from a combination of changes to the overall sediment budget (with sediment trapped behind dams on the Missouri River), land subsidence attributable in part to oil and gas extraction, sea level rise and increased erosion driven by more intense and frequent storms (Hiatt et al., 2019; Edmonds et al., 2023).

### 4.3 Ecosystem processes and functions in the Mississippi-Atchafalaya River Basin

To identify key processes (and their associated functions) in the MARB, we began by considering the ecosystem services it provides. We then worked backwards from those services to determine their supporting functions and processes.

Perhaps the most obvious ecosystem service provided by the MARB is a provisioning service: food production. The Upper Mississippi River Basin encompasses the Corn Belt, a globally important source of corn and soybeans. Downstream of the MARB, in the northern Gulf of Mexico, nutrient flows ultimately derived from the MARB help support economically important fisheries of shrimp, crab and red snapper. The relationships between upstream agriculture and downstream fisheries are complex; the high nutrient inputs that support high agricultural production also help to support primary productivity in the northern Gulf of Mexico (de Mutsert et al., 2016). However, those same nutrient flows also support development of an extensive hypoxic zone in the northern Gulf of Mexico (Rabalais and Turner, 2019). From a fisheries perspective, it has historically been challenging to separate the positive effects of nutrient additions from the negative effects of hypoxia. However, more recent work suggests that, at least for some species, the net effect of nutrients ultimately derived from agricultural production in the MARB is to decrease fish populations (Rose et al., 2018).

Our focus in this paper, however, is on the regulatory ecosystem services of the MARB (Table 2). Regulatory ecosystem services are those which maintain an environment conducive to life, by, e.g., maintaining and improving air and water quality. Regulatory ecosystem services are harder to value in economic terms than provisioning services; they have largely been under-valued because they are complex and largely invisible (Sutherland et al., 2018). We link each of these regulatory services and functions to associated MARB-related ecosystem processes in Table 2.

The key regulatory functions in the MARB, as identified by our group, are hydrologic regulation, energy regulation, biogeochemical regulation (primarily nutrient cycling), sediment regulation and water temperature regulation (Table 2). We excluded the regulatory functions of pollination or pest control here because, for most crops grown in the MARB, these functions are either not needed or are achieved using insecticides and herbicides. We did include habitat provision, which helps regulate biodiversity, in recognition of the key role played by biotic processes in many of the regulatory functions (Brodie et al., 2018; Leuzinger and Rewald, 2021).

### 4.4 Attributes of general river basin resilience in the Mississippi-Atchafalaya River Basin

Across a variety of ecosystems, connectivity, physical diversity (landscape composition, pattern and location), functional (biological) diversity and temporal variability have been identified as attributes of general resilience (McCluney et al., 2014; Grantham et al., 2019; Pelletier et al., 2020; Bullock et al., 2022). In river basins as large as the MARB, the form of these attributes and their relative importance may vary across ecosystem components and different regions of the MARB. For example, connectivity may include connections between isolated wetlands and headwater streams, and between the mainstem of the Mississippi River and its floodplain. We also recognize that the desired directionality of these attributes may vary in different settings and at different scales. As examples where increased connectivity may be beneficial we include: 1) re-establishment of river connections to floodplains and side channels, which can open up access to new habitat, facilitating fish reproduction (Beechie et al., 2023); 2) removal of passage barriers to facilitate migration of paddlefish and sturgeon (Tripp et al., 2019); and 3) dispersal and hence recovery of biological communities following disturbance (Oliver et al., 2015; Van Looy et al., 2019). However, in some cases increased connectivity may not be a good thing, as in the case of nutrient export from wetlands to streams, or for unique biological communities for which increased connectivity increases the risk of invasion by other species (e.g., Fuller and Death, 2018). The role of connectivity is likely to be scale dependent. Rolls etal. (2018) state that increasing connectivity leads to increased biodiversity at local scales, but decreased biodiversity at larger scales (i.e., overall biodiversity increases with increasing landscape fragmentation). Increased connectivity can also lead to increased sediment transport and export, which may not be desirable in certain settings (Fuller and Death, 2018).

Natural infrastructure can play an important role in increasing ecosystem resilience (Skidmore and Wheaton, 2022). Riparian buffers, nitrate-removal wetlands and reconnected floodplains provide ecosystem benefits across the MARB including improved water quality and flood risk reduction (Schilling et al., 2023). Grassland restoration (conversion of annual cropland to perennial vegetation) also represents a type of natural infrastructure that can benefit water quality and reduce flood risk, while also sustaining wildlife habitats and long-term carbon sequestration (Conant et al., 2017; Suttles et al., 2021; Schilling et al., 2023).

### 4.5 Conceptual ecosystem model

**FIGURE 6:**
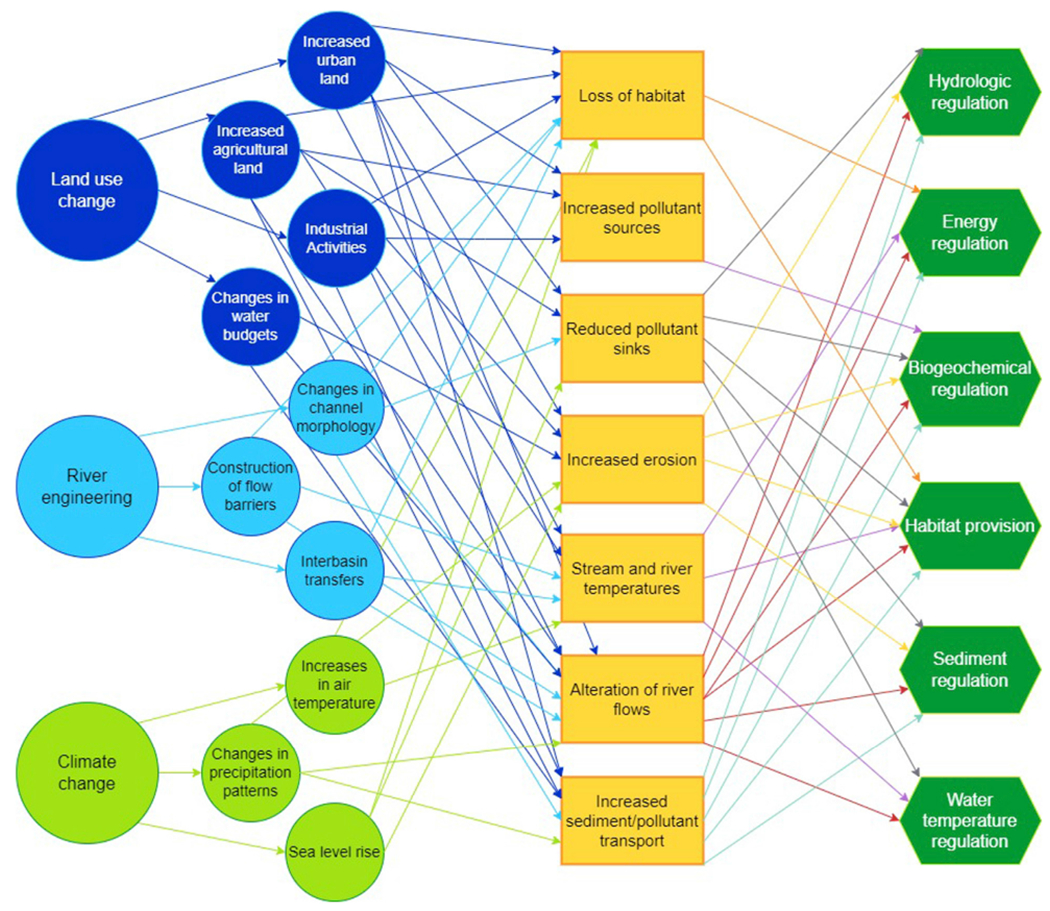
Conceptual model of the relationships between drivers, stressors and ecosystem functions in the Mississippi-Atchafalaya River Basin ecosystem. Drivers (taken from Table 1) are shown as circles, stressors are shown as rectangles and the affected ecosystem functions (taken from Table 2) are shown in hexagons.

Based on our identification of the most important stressors (Table 1), as well as the most important ecological processes and functions (Table 2), we propose a simple conceptual model of the MARB (Figure 6) demonstrating how these stressors affect ecological processes and functions at river basin scale. For example, land use change in the form of an increased percentage of agricultural land in the MARB leads to loss of wetlands, which in turn affects multiple ecological functions (reduced pollutant sinks, changes in water budget and loss of habitat). Our conceptual model shows how each of the major stressors (land use change, river engineering and climate change) affects multiple ecosystem functions. Although our model is conceptual and based on our collective experience in the MARB, it is congruent with the results of more formal analyses using Structural Equation Models, boosted regression trees, neural networks, or similar approaches to understand causes of ecosystem degradation in this system (e.g., (Schmidt et al., 2019; Mengistu et al., 2020; Waite et al., 2021).

## 5 Potential management actions to improve ecosystem health in the Mississippi-Atchafalaya River Basin

Where our generalized framework for improving ecosystem health (Figure 3) identifies three approaches to be used in combination to improve ecosystem health, our conceptual model for the MARB ecosystem (Figure 6) allows us to be more specific about the stressors that need to be reduced and the ecological functions that need to be enhanced. Combining these insights with attributes of general river basin resilience in the MARB (described above) allows us to identify a suite of potential management actions to improve ecosystem health (Table 3 and Supplementary Table S2). We have organized these potential actions according to the approaches called for in our generalized framework for improving ecosystem health: reducing stressors, restoring or enhancing ecological functions, and increasing resilience. Table 3; Supplementary Table S2 are intended to be illustrative rather than prescriptive, but they illuminate the diverse scope of actions that could improve ecosystem health.

As we noted earlier, climate change is a global issue, which cannot be completely mitigated at the scale of the MARB. There are, however, opportunities to reduce greenhouse gas emissions within the MARB and we have included them here for completeness. Equally important, actions to increase carbon sequestration in perennial vegetation within the MARB are likely to increase resilience at small to medium scales. However, it is the other actions listed here—minimizing the effects of land use change and river engineering, restoring and enhancing ecosystem function, and increasing the attributes of river basin resilience—that may have a greater effect on mitigating the effects of climate change on the MARB.

## 6 Indicators of ecosystem health for the Mississippi-Atchafalaya River Basin

Based on our generalized framework for improving ecosystem health (Figure 3), and our conceptual ecosystem model (Figure 6) for the MARB, we hypothesize that it will be possible to track changes in ecosystem health by quantifying changes in MARB-specific stressors, ecosystem functions, and attributes of general river basin resilience.

To track changes in stressors, we have focused on landscape indicators—quantitative metrics that describe the compositional and spatial aspects of landscapes—as these have been widely used to track changes in stressors at scales ranging from small stream watersheds to large river basins (see, e.g., Aho, Flotemersch et al., 2016; Aho, Flotemersch et al., 2020; Comte et al., 2022). These indicators generally categorize the extent and/or intensity of the stressor, both of which have been shown to affect water quality and biota (Waite, 2013; Davis et al., 2015).

To track changes in ecosystem function, we reviewed the literature on—and talked with practitioners of—direct monitoring of processes, such as ecosystem metabolism, organic matter decomposition and nutrient cycling. Although the technical ability to measure ecosystem metabolism has greatly advanced in recent years (Ferreira et al., 2020), such monitoring remains limited, especially across large spatial scales, because it is difficult to ensure comparability of measurement techniques (Brosed et al., 2022). Additionally, interpretation of site-specific results in terms of ecosystem health remains challenging (Mejia et al., 2018) and high spatial variability between sampling sites makes it difficult to synthesize results from specific sites to entire watersheds (Mancuso et al., 2022). For this reason, we decided to seek an alternative approach to monitoring ecosystem function, and turned instead to reviewing studies that have shown strong statistical relationships between landscape composition and pattern and various ecological processes (Qiu and Turner, 2015; Duarte et al., 2018; Qiu, 2019; Metzger et al., 2021). Emerging science seeks to explain these relationships more mechanistically in terms of dynamically variable temporal and spatial connectivity across different aquatic systems (Leibowitz et al., 2018; Evenson et al., 2021). We, therefore, decided to focus on indicators that emphasize the spatial arrangement of sites in networks, and the connections (or lack of connections) between them (Kuemmerlen et al., 2019).

Landscape indicators are likewise routinely used to measure the key attributes of resilience: diversity of flora and fauna and connectivity of landscape and hydrology (Allen et al., 2016; Bouska et al., 2019). We chose to also include the percentage of the landscape in perennial native vegetation as an indicator of river basin resilience, following the work of Oliver et al. (2015) who noted that perennial native vegetation promotes genetic diversity and functional redundancy, both of which increase the resistance of ecosystem functions to change.

In selecting indicators to track, we have been sensitive to challenges of spatial scale and cost. Local communities may be interested in changes in ecosystem health at the scale of individual sites and small watersheds. However, Federal and State agencies, which are likely to provide the bulk of funding for improvement in ecosystem health, target much larger spatial extents (e.g., landscapes, large watersheds/river basins). It is important, therefore, to identify indicators that can be measured at both local and larger scales, and we have attempted to do so. In addition, recognizing that funding to support monitoring is always limited, we have focused on the opportunity to use data obtained from maps, GIS coverages and remotely sensed images as a relatively low-cost way of gathering data across large areas.

Table 4; Supplementary Table S3 show the set of potential indicators that we propose could be used to track changes in ecosystem health in the MARB. Where possible, we selected indicators for which change over time could be tracked using remote-sensing approaches. In other cases, remote sensing approaches to data collection are in development, but are not yet deployed at scale, or not yet deployed in the MARB.

The indicators shown in Table 4 are examples of “leading” indicators (sensu R. K. Hall et al., 2014; E. S. Hall et al., 2019; Ota et al., 2021) that can be used to predict future changes in ecosystems, while “lagging” indicators (such as measurements of water quality and species abundance) are retrospective, showing how the ecosystem has responded to past changes in leading indicators. We suggest that ecosystem managers could benefit from a more comprehensive approach to monitoring that includes both leading and lagging indicators of ecosystem health. We further suggest that this can be done at relatively low cost, by supplementing current site-level monitoring of “lagging indicators” with remotely sensed data on “leading” landscape-level indicators of the type shown in Supplementary Table S3.

**FIGURE 7:**
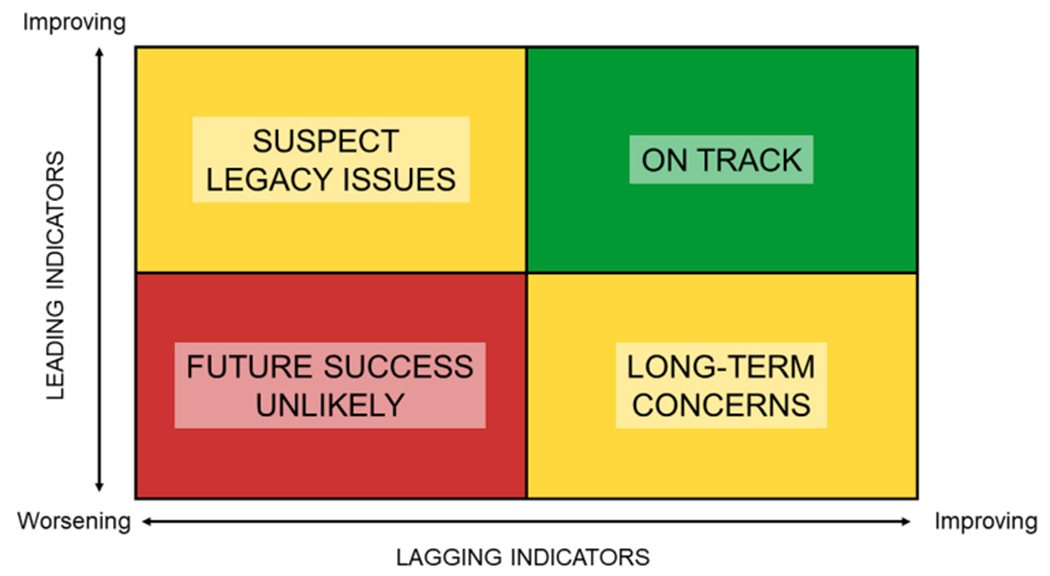
Using leading and lagging indicators of ecosystem health in an adaptive monitoring scheme. By comparing trends in leading and lagging indicators, ecosystem managers can assess the effect of management actions. Where both leading and lagging indicators are improving, as in the top right quadrant, management actions are having the desired effect. Where leading indicators are improving but lagging indicators are stable or worsening, as in the top left quadrant, managers might investigate the potential for legacy effects (as often seen in water quality monitoring). Where leading indicators are worsening but lagging indicators are improving, as in the bottom right quadrant, there may be cause for long-term concern if stressor levels exceed a threshold for response. Finally, where both leading and lagging indicators are worsening, as in the bottom left quadrant, management efforts are proving inadequate to address the fundamental drivers of ecosystem degradation, and future success is unlikely unless this is addressed.

This combination of approaches would provide better-informed management decisions, by improving our understanding of how the ecosystem works and allowing us to test whether management actions are having the desired effect (Marshall and Negus, 2019; Negus et al., 2020). It would enable a hypothesis-based, adaptive monitoring and management system that can be used to test the effects of management actions and inform any needed changes (Lindenmayer and Likens, 2010), as illustrated in Figure 7.

The judgment of whether changes in a specific indicator correspond to improving or declining ecosystem health must be made in context. For example, in the upper portion of the MARB historic erosion from agricultural lands has had the effect of choking many stream and river channels with sediment; in this case, decreased sediment loads would represent an improving condition. In the Mississippi River Delta, on the other hand, retention of sediment behind the dams on the Missouri River has led to sediment starvation of coastal wetlands and their increased vulnerability to sea level rise; in this case, an increase in sediment loads would represent an improving condition. Likewise, while artificial drainage in the upper, agricultural portion of the MARB has led to increased, erosive flows, in the lower portion of the MARB groundwater abstraction has lowered streamflows with effects on aquatic life; in the former case, improving conditions might be represented by decreased flows, whereas in the latter case increased flows might be more desirable.

An ecosystem of interest (in this case, a small or large watershed, a regional sub-basin, or the entire MARB) can be placed into one of the quadrants of Figure 7 depending upon whether leading indicators are worsening or improving and by whether lagging indicators are worsening or improving. In the top right quadrant, for example, both leading and lagging indicators would show improvement, from which we could infer that management actions are on track to lead to desired ecosystem conditions.

Conversely, if leading indicators do not show improvement, this can serve as an early warning system that fundamental challenges to ecosystem health are not being addressed (bottom left and right quadrants of Figure 7). An example of this situation may be occurring in the Upper White River watershed in Indiana. The most recent water quality trend report from the USGS (data collected through 2020) showed improved water quality for all four measured constituents at one gage and improvement in nitrogen/nitrate at all three gages (Koltun, G.F., 2023). However, this is a rapidly urbanizing watershed and in the 3 years since those water quality measurements were taken at least 103 acres of wetlands were removed under Indiana’s stream and wetland mitigation *in lieu* fee program, which sells wetland mitigation credits for unavoidable impacts to wetlands (Indiana Dept of Natural Resources, 2023). The seeming improvement in lagging indicators (water quality) coupled with the decline in leading indicators (wetland acreage) suggests that this watershed would be placed in the bottom right quadrant of Figure 7, meaning there is cause for concern that current “success” as measured by water quality will not be sustained in the long term.

In the MARB and across the U.S., decades of effort to reduce nutrient loadings from agriculture have not resulted in the expected improvements in stream and river water quality (Secchi and Mcdonald, 2019; Stackpoole et al., 2019; Stets et al., 2020). The analysis by Stackpoole et al. (2021) of trends in phosphorus (P) concentrations in U.S. rivers showed that, in many agricultural watersheds, P balances—a leading indicator of nutrient pollution—decreased. However, those trends did not translate into consistent water quality improvements, and P export actually increased in many of these watersheds. The improvements in leading indicators coupled with the stasis or worsening of lagging indicators (P export) suggest that those watersheds would be placed in the upper left quadrant of Figure 7, where legacy nutrient issues obscure the expected effects from management efforts. In such cases, a focus on lagging indicators alone may wrongly lead to the conclusion that management is ineffective.

## 7 Discussion

Our approach to assessing ecosystem health is aligned with several other recent efforts. In the Colorado River Basin, Paukert et al. (2011) and Comte et al. (2022) have undertaken a high-resolution, basin-wide spatial mapping of threats based on landscape-level indicators and they have shown that threat levels correspond to indicators of ecological condition, such as biota, water quality and flow modification. Also, a series of studies using the Index of Watershed Integrity (Flotemersch et al., 2016) have emphasized the relationship between ecosystem stressors and ecosystem condition (Kuhn et al., 2018; Thornbrugh et al., 2018; Johnson et al., 2019) and applications of this approach have been developed for ecosystem managers in Alaska (Aho et al., 2020a) and Western Balkans (Aho et al., 2020b). Harwell et al. (2019) have likewise used a stressor-based approach to develop a set of EcoHealth metrics for the Gulf of Mexico. Our study takes threat/stressor mapping a step further by developing a conceptual ecosystem model linking stressors to ecosystem responses and using this model to identify additional opportunities to improve ecosystem health by reducing stressors, improving ecosystem function, and increasing river basin resilience.

All of these approaches are fundamentally based on a Driver - Pressure (Stressor)—State—Impact—Response framework designed to help managers diagnose the causes of ecosystem problems, and thus identify appropriate treatment (Smeets and Weterings, 1999). They stand in contrast to more public-facing approaches such as the Chesapeake Bay Report Card (https://ecoreportcard.org/report-cards/chesapeake-bay/publications/), the System-wide Ecological Indicators for Everglades Restoration reports (https://www.evergladesrestoration.gov/s/2020-systemwide-ecological-indicators-031821.pdf) and the America’s Watershed Initiative Mississippi River Watershed Report Card (https://americaswatershed.org/wp-content/uploads/2020/12/AWI-Report-Card-2020.pdf), all of which focus on ecosystem condition. In drawing this distinction, we intend only to point out that the information needed by managers for decision-making may be quite different from the information that the public needs to measure the success of management efforts. Both types of information are important, but they are used in different contexts.

In applying this conceptual framework, the nature of the stressor-response curves is important but often highly uncertain. Nonetheless, management choices must be made in the context of this uncertainty. In conceptualizing the relationship between stressors and ecosystem response, we have not attempted here to assess shapes of all stressor-ecosystem response curves, in particular whether the curves show threshold behavior in which a small increase in stressor creates a large change in response (Larned and Schallenberg, 2019); if so, managers must determine trigger values at which changes in management are needed to avoid crossing a threshold. There is some evidence that the largest changes in water quality occur above certain (threshold) values of agricultural and urban land use (Wagenhoff et al., 2017; Snyder and Young, 2020), but more work remains to be done to identify potential thresholds. Hillebrand et al. (2020) in a cautionary note, suggested that most studies lacked the statistical power to discern these thresholds, yet their existence can dominate watershed behavior and generate unexpected phenomena. Further complicating efforts to identify ecological thresholds in ecosystems is that aquatic ecosystems are subject to multiple, often synergistic, stressors (Waite et al., 2021; Carrier-Belleau et al., 2022). The determination of thresholds (and response curves in general) is a priority for scientific investigation. The use of such ecological thresholds in decision-making is still further complicated by the fact that every jurisdiction across a river basin has the ability to set a different threshold.

We urge broader discussion within the management community regarding pathways to improved ecological health, in particular whether to emphasize stressor reduction or enhancement of ecological function. In a regulatory setting, stressor reductions are likely to be more measurable and thus more certain; Schallenberg (2021) provides three examples of using well-constrained stressor-response curves to set nutrient loading limits (stressor reduction targets) in lakes in New Zealand, and the Total Maximum Daily Load program under the U.S. Clean Water Act likewise focuses on stressor (pollutant load) reduction. However, others have argued for a more holistic approach that incorporates efforts to restore ecological function through habitat restoration, particularly in riparian areas (Hall et al., 2014) and there is likely to be value in incorporating both approaches (Cook et al., 2015).

## 8 Conclusion

We present a generalized approach for improving ecosystem health by reducing ecosystem stressors, and thereby restoring and enhancing ecosystem function and increasing resilience in large river basins. We illustrate how this approach can be applied to a large river basin, the MARB of the central U.S., by developing a conceptual ecosystem model that connects stressors to ecosystem responses. From this model, we derive a set of landscape-scale indicators of ecosystem health. We suggest that expansion of current monitoring programs to incorporate these leading indicators would allow testing of the conceptual ecosystem model and the effectiveness of the management actions derived from it. We further suggest that combined monitoring of leading and lagging indicators could help managers both evaluate the success of past actions and prioritize future actions.

Although our ecosystem model and ecological indicators are specific to the MARB, we suggest that our approach to developing these tools is broadly applicable to other large river basins, and potentially to other ecosystem types. In particular, we suggest that the main categories of stressors identified in the MARB—which themselves are ultimately driven by land use change, river engineering and climate change—affect river basins across the globe, and that our proposed management actions and ecological indicators may be broadly transferable.

Finally, in assessing the health of a specific ecosystem, it is important to define the ecosystem very broadly in terms of ecosystem components. In this paper, we chose to look at both terrestrial and aquatic components in order to capture all potential stressors and responses. Such comprehensive scientific and geographic perspectives are foundational to understanding the linkages between landscapes and riverscapes (Allan, 2004). Further, in managing ecosystems for change, complexity should be considered. Specifically, in ecosystems of the size of the MARB, there is no “one-size-fits-all” approach to improving ecosystem health. In the face of global change, increasing ecosystem complexity (via structural heterogeneity, flow regimes, connectivity, biodiversity) is the best approach for enhancing ecosystem function and resilience (Bullock et al., 2022).

Introducing complexity to the landscapes and riverscapes of the MARB will not be easy. Hundreds of years of human history have moved the region in the opposite direction, simplifying complexity in the service of efficiency. However, growing awareness of climate change-related disasters may prompt governments and the private sector to place more emphasis on the need for river basin resilience to risks at a variety of scales (Hynes et al., 2020), which could increase the willingness to embrace system complexity.

## Supplementary Material

Supplement1

## Figures and Tables

**TABLE 1:** Key drivers of ecosystem degradation in the Mississippi-Atchafalaya River Basin.

Driver category	Key driver	Examples within MARB
1. Land use change	Increased percentage of agricultural land and associated loss of native vegetation	*Conversion of prairies, wetlands and forests to cropland*
	Increased agricultural intensity (increased anthropogenic inputs and/or drainage modification per unit of agricultural area)	*Increased nitrogen (N) inputs for crop production*
		*Increased pesticide inputs for crop production*
		*Artificial drainage of cropland in the Upper Mississippi River Basin*
		*Groundwater withdrawals for irrigation in the Lower Mississippi River Basin*
	Increased percentage of urban land	*Conversion of agricultural land to urban and suburban uses*
	Increased urban intensity	*Increased extent of impervious surfaces*
		*Increased inputs of toxics and emerging constituents of concern*
		*Groundwater withdrawals for urban use*
	Increased industrial activities	*Land subsidence in the Mississippi River Delta due in large part to oil and extraction*
	Changes in local and regional water budgets	*Wetland loss, prairie loss, forest loss*
		*Artificial drainage of cropland in the Upper Mississippi River Basin*
		*Groundwater withdrawals for irrigation and urban use*
2. River engineering	Changes in channel morphology	*Stream channelization in the Upper Mississippi River Basin*
		*Levee construction in the Lower Mississippi River Basin and Mississippi River Delta*
		*Construction of wing vanes and other in-river structures in the Lower Mississippi River*
		*Sediment starvation of coastal wetlands in the Mississippi River Delta*
	Construction of flow barriers	*Dams and impoundments on the mainstem Mississippi and Missouri Rivers and on the Illinois and Ohio Rivers in the Upper Mississippi River Basin*
	Interbasin transfers	*Water transfers from the MARB to states in the Western U.S.*
3. Climate change	Increased mean annual near-surface air temperature	*Temperatures have increased 0.8 °C since 1850*
	Intensification of hydrologic cycle	*Both the frequency and intensity of rainfall and droughts are increasing*
	Relative sea level rise (RSLR)	*Present-day RSLR rates in coastal Louisiana are among the highest in the world at ~12 mm per year*

**TABLE 2:** Key regulatory ecosystem services/functions and associated processes in the Mississippi-Atchafalaya River Basin (adapted from Flotemersch et al., 2016).

Service/Function	Description	Associated ecosystem processes
Hydrologic regulation (including water supply regulation and natural hazard [flood] regulation)	Maintenance of the natural timing, pattern, supply, and storage of water flows (including groundwater flows)	Surface runoff
		Groundwater flow
		Evapotranspiration
		Water retention
Energy regulation	Maintenance of the natural flows of energy among ecosystem compartments, including, e.g., trophic cascades	Primary production
		Secondary production
		Metabolism
		Decomposition
Biogeochemical regulation (including water purification and climate regulation)	Maintenance of the natural fluxes of chemical elements and compounds among ecosystem components	Nutrient cycling
		Nutrient retention and removal
		Carbon cycling
		Carbon sequestration
		Pesticide degradation
		Waste treatment
Sediment regulation (including erosion protection)	Maintenance of the volume and size composition of inorganic particles stored or transported through streams, lakes, wetlands, or estuaries	Erosion
		Sediment transport
		Sediment deposition and retention
Water temperature regulation	Maintenance of the full range of landscape features required to maintain water temperatures that support the natural diversity and abundance of biota	Riparian shading
		Hyporheic discharge
		Stream discharge, urban runoff and agricultural return flows
Habitat provision	Presence and maintenance of the full range of natural features needed to maintain the natural diversity and abundance of biota	Processes associated with biota
		Primary production
		Secondary production
		Metabolism
		Decomposition
		Nutrient cycling
		Nutrient retention and removal
		Carbon cycling
		Carbon sequestration
		Pesticide degradation

**TABLE 3:** Potential management actions to improve ecosystem health in the Mississippi-Atchafalaya River Basin.

Management goal	Examples of management actions
**Reduce stressors**	
Minimize effects of land use change	Create land protection programs and mitigation requirements to minimize loss of existing forests, grasslands and wetlands
	Develop regulatory approaches or voluntary programs to reduce unavoidable effects of land use change (e.g., use of agricultural and urban Best Management Practices to manage nutrient inputs and stormwater flows)
Minimize effects of river engineering	Provide policy and financial incentives for river restoration
	Modify design and operation of dams and levees to restore more natural flow regimes
	Provide policy to limit interbasin transfer of water
Mitigate climate change	Create land protection programs to minimize loss of existing carbon stocks
	Use voluntary or regulatory approaches to reduce emissions of greenhouse gases
	Create incentives to promote the sequestration of carbon in perennial vegetation
**Restore and enhance ecosystem functions and processes**	
Improve nutrient cycling	Create incentives for the use of whole-farm and multi-year nutrient budgeting practices that increase soil organic matter and soil microbial communities, controlled drainage, tailwater recycling on irrigated fields, and integration of crop and livestock production, all of which support improved nutrient management
Improve nutrient retention and removal	Promote restoration of ponds, floodplain and non-floodplain wetlands, and restored drainage ditches and streams, all of which can serve as nutrient sinks
Restore sediment retention in uplands	Promote restoration of ponds and non-floodplain wetlands to store sediment in the landscape
Restore sediment flow regime in river channels	Support reconnection of river channels and floodplains (e.g., oxbow lake restoration, floodplain restoration, sediment diversions) and create sediment bypass tunnels on dams (Lower Missouri River tributaries) to restore sediment regimes
Increase water storage in landscape	Promote water-harvesting techniques (e.g., bunds, ponds, percolation tanks) on farmland and restore wetlands (floodplain and non-floodplain) to create water storage
Increase groundwater recharge	Provide incentives for grassland and forest restoration, and for the development of infiltration basins
Restore flow variability in streams and rivers	Re-operate dams for flow variability, especially flood pulses; reconfigure/setback levees; explore opportunities to restore a more natural flow regime by, e.g., acquiring additional water rights
Restore thermal buffering for streams and rivers	Encourage appropriate riparian planting; restore hyporheic connections to groundwater via stream restoration (e.g., use of pools and riffles, log dams)
**Increase general river basin resilience**	
Increase biodiversity at all scales	Create incentives for the following: crop diversification (e.g., extended rotations and more diverse cropping systems); re-integration of crop and livestock production; landscape diversification (e.g., creation and/or restoration of hedgerows, prairie strips, woodlots, wetlands, and floodplain forests-including the use of diverse vegetation types in all of these); management of invasive species
Increase terrestrial habitat connectivity to facilitate species dispersal	Prioritize conservation funding to connecting existing habitat patches
Increase aquatic habitat connectivity to facilitate species dispersal	Remove artificial barriers or create passage structures through or around barriers; manage flow regimes for increased connectivity
Create thermal refugia (aquatic)	Support the creation of riparian buffers; support stream restoration that enhances hyporheic exchange

**TABLE 4:** Potential indicators to track ecosystem health.

Management goal	Potential indicators
**Reduce stressors**	
Minimize effects of land use change	Changes in % of forest, grassland and wetland cover at county or small watershed scale
	For nutrients: changes in anthropogenic N and P inputs at county scale
	For toxins (e.g., pesticides): changes in amounts of pesticides applied
	Changes in extent of winter vegetative cover (either NDVI or fractional green vegetation cover) and crop residue
	Changes in length of tile drainage
	Deviations from natural flow conditions (assessed using streamflow data from USGS gauge stations compared to estimated natural flow conditions)
	Changes in volume of groundwater withdrawals
	Changes in volume of inter-basin water transfers
Minimize effects of river engineering	Changes in channel sinuosity (e.g., due to ditching of headwater streams)
	Minimize impacts of river engineering Changes in area of aquatic habitat
	Changes in area of floodplain disconnected by levees
	Changes in number of stream and river flow barriers
Mitigate climate change	Changes in number of snow cover days
	Changes in regional atmospheric concentrations of CO_2_, CH_4_ and N_2_O
	Changes in amount of carbon uptake in tree/shrub biomass
**Restore and enhance ecosystem functions and processes**	
Improve nutrient cycling	Changes in losses of N and P to air and water, quantified by changes in N and P balance
Improve nutrient retention and removal	Changes in area of hydrologically connected wetlands Changes in area of hydrologically connected floodplains
Restore sediment retention and sinks	Changes in area of hydrologically connected floodplains
	Changes in annual volume of sediment accreted in and lost from the Mississippi River Delta
Increase water storage in landscape	Changes in water storage capacity in ponds and depressional wetlands; changes in potential water storage in reconnected floodplains
Increase groundwater recharge	Changes in land cover coupled with remotely sensed data on evapotranspiration and soil moisture
Increase flow variability in streams and rivers; restore functional	Changes in multiple aspects of streamflow, e.g., floodplain inundation frequency, frequency and magnitude of peak flows during specific seasons; stability of base flows
Restore thermal buffering for streams and rivers	Changes in extent of riparian vegetation; changes in hyporheic discharge
**Increase river basin resilience**	
Increase biodiversity at all scales	Changes in various vegetative diversity indices (or biodiversity indices if amenable to data collection)
Increase terrestrial habitat connectivity to facilitate native species migration	Changes in connectivity index at multiple scales
Increase aquatic habitat connectivity to facilitate native species movement	Changes in hydrologic connectivity index at multiple scales
Increase % watershed in perennial vegetation	Changes in % perennial cover

## Data Availability

The original contributions presented in the study are included in the article/Supplementary Material, further inquiries can be directed to the corresponding author.
