# Supplementary material for: Improving ecosystem health in highly altered river basins: a generalized framework and its application to the Mississippi-Atchafalaya River Basin
Source: Front Environ Sci. Author manuscript; Available in PMC 2025 Feb 22. (PMC10953731; doi:10.3389/fenvs.2024.1332934)
Supplement: Supplement1 [file NIHMS1973944-supplement-Supplement1.pdf]

Supplementary Material

Improving ecosystem health in highly altered river basins: a generalized framework and its application to the Mississippi-Atchafalaya River Basin

Eileen L. McLellan, Kelly M. Suttles,* Kristen L. Bouska, Jamelle H. Ellis, Joseph E. Flotemersch, Madison Goff, Heather E. Golden, Ryan A. Hill, Tara R. Hohman, Shamitha Keerthi, Richard F. Keim, Barbara A. Kleiss, Tyler J. Lark, Bryan P. Piazza, Alisha A. Renfro, Dale M. Robertson, Keith E. Schilling, Travis S. Schmidt, Ian R. Waite

*** Correspondence:** Corresponding Author: ksuttles@edf.org

Supplementary Tables

Table S1. Key drivers of ecosystem change in the Mississippi-Atchafalaya River Basin (MARB).

**Driver category.**	**Key driver.**	**Examples within MARB.**
1. Land use change	Increased percentage of agricultural land and associated loss of native vegetation	*Conversion of prairies and wetlands to cropland.*<br>In the late 2000s and early 2010s, approximately 400,000 hectares (ha) of grasslands and other natural landcovers were converted to cropland each year (Lark et al., 2020).<br>Only about 4% of tall-grass prairie remains from its historic extent. Over a five-year period (2008-2012) roughly 77% of new cropland was converted from prairie (2.3 million ha) (Bajgain et al., 2018).<br>In 2010-2013, almost 30% (530,000 ha) of expiring Conservation Reserve Program (CRP) land returned to agricultural production in the U.S. Midwest. CRP grasslands and wetlands converted were 360,000 ha and 53,000 ha, respectively (Morefield et al., 2016).<br>By 2017 the total area of cropland increased by 3.5 million hectares from 2007. Conversion of non-cropland-to-cropland (former grasslands and land leaving the Conservation Reserve Program) increased total U.S. cropland by 3.3% (Zhang et al., 2021).<br>Thirty million hectares of wetlands were drained for agriculture from 1901-2000 in the Mississippi River Basin (Mitsch and Day, 2006).<br>In the Midwest 10 million ha of closed canopy forest were converted to agriculture over the last 200 years (Hanberry and Abrams, 2018).
	Increased agricultural intensity (increased anthropogenic inputs and/drainage modification per unit of agricultural area).	*Increased nitrogen (N) inputs for crop production.*<br>In U.S. agriculture, synthetic nitrogen fertilizer use per unit of cropland increased 4-fold between 1960 and 2015, from 19 to 90+ kg N/hectare (US EPA, 2022). U.S. Environmental Protection Agency (EPA) Regions 5 and 7 in the Corn Belt had the highest total use of N fertilizer, with annual use of over 2.4 million metric tons each in 2017-18.<br>*Increased pesticide inputs for crop production.*<br>In the Midwest, agricultural pesticide use was at least 2.5 times higher (49 kg km^{-2}) compared to other regions (Northeast, South, West, and Pacific, 3 to 21 kg km^{-2}) (Stackpoole et al., 2021).<br>Pesticide use in U.S. agriculture increased more than 3-fold between 1960 and 1981 (from 89,000 to 287,000 metric tons of active ingredient), with slight decline by 2008. Since 1980, over 90% of the corn, soybean, and cotton crop area is treated with herbicides (Fernandez-Cornejo et al., 2014).

Driver category.	Key driver.	Examples within MARB.
1. Land use change	Increased agricultural intensity (increased anthropogenic inputs and/drainage modification per unit of agricultural area).	*Artificial drainage of cropland in the Upper Mississippi River Basin.*<br>By 2004 the area of artificially drained land in Iowa increased by about 1.1 million hectares from 2.5 million hectares in the late 1920's, which accounts for 99% of the poorly drained soils in the state (Boland-Brien et al., 2014; Schilling et al., 2012).<br>As of 2017, 84% of tile-drained croplands in the United States are found in six Midwestern states (18.79 million ha) (Valayamkunnath et al., 2020).<br>Total U.S. cropland area under tile drainage increased from 20 to 23 million hectares between 2012 and 2017 (USDA - National Agricultural Statistics Service, 2019). However, fluctuations in reported total tile-drained area are not new – as the Census of Agriculture reported 21, 18, 24, and 17 million hectares of drained land (sub-surface tile plus surface drained) already in 1920, 1930, 1969, and 1974, respectively (Jaynes and James, n.d.). In recent years, many farmers have replaced or added onto older drainage systems (University of Illinois Extension, 2023).<br>*Groundwater withdrawals for irrigation in Lower Mississippi River Basin.*<br>Four million ha of cropland in the Lower Mississippi River basin depend on the Mississippi River Valley alluvial aquifer to provide 90% of irrigation water, with Arkansas using about 70% of that water (Reba and Massey, 2020) (1/2 of it for rice production (Reba et al., 2017)). Irrigated land increased 12-fold in the state of Arkansas and doubled in Louisiana between 1950 and 2017 (Reba and Massey, 2020).
	Increased percentage of urban land.	*Conversion of agricultural land to urban and suburban uses.*<br>Coastal wetland migration is constrained by development adjacent to the wetlands along the Gulf Coast in the Atchafalaya/Vermilion Bays in Louisiana (Borchert et al., 2018).<br>Six rural watersheds around Kansas City lost an average of 28% (range 20-41%) of grassland/agriculture areas to urbanization from 1992 to 2014 (Zubair et al., 2019).
	Increased urban intensity.	*Increased extent of impervious surfaces.*<br>In Indianapolis, IN 3.65% of the total land cover changed from 2001 to 2006. Of that area 51% changed from crop land to developed land, 31% changed from a lower level of developed land to a higher level of developed land, and 3.6% was changed from forest to developed (Jiang et al., 2015).

**Driver category.**	**Key driver.**	**Examples within MARB.**
1. Land use change	Increased urban intensity.	*Increased extent of impervious surfaces.*<br>In the St. Louis Metropolitan area, impervious surfaces increased by 11.2% from 2001 to 2011 leading to a 125-175% increase in peak discharges (Hu and Shrestha, 2020).<br>In Missouri rural areas were rapidly converted to impervious surfaces during the 1990s (481 km^2) (Zhou et al., 2012).<br>*Increased inputs of toxics and emerging constituents of concern.*<br>Fecal coliform concentrations in St. Tammany parish, Louisiana are strongly correlated with increased urbanization, which increased 218% from 1982 to 2000 (Carstens and Amer, 2019).<br>More pharmaceuticals, personal care products, and pesticides were detected at two Midwestern U.S. National Parks with greater urban influences than at the two more remote parks (Elliott and VanderMeulen, 2017).<br>Twenty-seven neuropsychiatric and eight illicit drugs were detected in the Bee Creek receiving waters from a Kentucky wastewater treatment plant. Of these methamphetamine and amphetamine had the highest reported per-capita consumption rates in the USA (Skees et al., 2018).<br>Microplastics have been found in fish distributed widely throughout the main stem of the Mississippi River (Gad et al., 2023).<br>*Groundwater withdrawals for urban use*<br>Increased urban water use is depleting groundwater near Memphis, causing interstate political conflict (Abrams and Markus, 2021).
	Increased industrial activities.	*Land subsidence in the Mississippi River Delta due in large part to oil and extraction.*<br>Naturally high subsidence rates in coastal Louisiana, on average 9 mm yr^{-1}, are no longer offset by sediment from the River (Nienhuis et al., 2017).
	Changes in local and regional water budgets.	*Wetland loss, prairie loss, forest loss.*<br>Over 10 million ha of forested wetlands covered land between the Ohio River and the Gulf of Mexico, but 75% of them have been lost to conversion to agriculture and flood control projects (Berkowitz et al., 2020). Conversion of forested wetlands to other land uses increases runoff, decreases evapotranspiration and alters the timing, extent,

		and volume of downstream flood events (Berkowitz et al., 2020).<br>Extensive land cover changes can alter the mesoscale atmospheric environment, including the severity of thunderstorms, excessive precipitation, and quick onset of droughts due to a shift in the growing season evapotranspiration fluxes (Bajgain et al., 2018).<br>Modeled wetland loss results in a change in flood magnitude (e.g., 50-year flood vs. 100-year flood) in the Prairie Pothole region (Golden et al., 2021).
1. Land use change	Changes in local and regional water budgets.	*Artificial drainage of cropland in the Upper Mississippi River Basin.*<br>84% of tile-drained croplands in the United States are in six Midwestern states (18.79 million ha) (Valayamkunnath et al., 2020); these significantly alter runoff volume, baseflow, groundwater travel times, the concentration of in-stream nitrate, and the timing and shape of the hydrograph (Valayamkunnath et al., 2020).<br>Sites with extensive ditch networks and tile drain systems had higher magnitude floods in the upper Midwest (Prestegaard et al., 1994).<br>*Groundwater withdrawals for irrigation and urban use.*<br>From 2000 to 2015 groundwater withdrawals increased 40% in the Mississippi River Valley alluvial aquifer (Reba and Massey, 2020). Measurements from 2012 indicate that Arkansas was pumping twice as much as the calculated sustainable yield and by 2050 the eastern part of the state is projected to have a groundwater supply gap of 27 Mm^3 d^{-1} (Reba and Massey, 2020). Streamflow can be diminished when the lower water levels in the aquifer become disconnected from overlying streams and rivers (Reba and Massey, 2020).<br>*Stream channelization in Upper Mississippi River Basin.*<br>Within the last 150 years in Illinois, it is estimated that 25% of all streams and almost all headwater streams have been channelized (Becker and Peterson, 2022).
2. River engineering.	Changes in channel morphology.	*Levee construction in the Lower Mississippi River Basin and Mississippi River Delta.*<br>There are nearly 38,657 km of levees in the Lower Mississippi basin, 35,374 km in the Upper Mississippi basin and 12,216 km in the Ohio River basin (Knox et al., 2022).<br>*Construction of wing vanes and other in-river structures in the Lower Mississippi River.*<br>In free-flowing reaches of the Mississippi River more than 847 km of dikes have been constructed to prevent meandering and promote scour in the primary channel (Schramm and Ickes, 2013). By adding notches to the dikes, water flows through at lower velocities creating more diverse aquatic habitats (Schramm and Ickes, 2013). Chevron dikes, used to direct flow to maintain the

		navigation channel, also create improved habitat (Schramm and Ickes, 2013) and in one study increased physical-aquatic-habitat diversity by 8-35% (Remo et al., 2013).<br>Revetments have reduced the sediment supplied to the River from bank caving by 96% since 1931 (Murray and Biedenharn, 2022).
2. River engineering.	Changes in channel morphology.	*Sediment starvation of coastal wetlands in Mississippi River Delta.*<br>Subsurface resource extraction, levees and upstream dams have all contributed to land loss in coastal Louisiana. At its peak, subsurface resource extraction doubled the rate of land loss from to 14 km^2 yr^{-1} (Edmonds et al., 2023).<br>Without sediment input coastal Louisiana is expected to lose an additional 10,000-13,500 km^2 of land by 2100 due to sea level rise and subsidence (Xu et al., 2019).<br>Dams on Missouri River: 200 Mt y^{-1} of fine sediment is trapped behind dams built on the Lower Missouri River in the 1950s and 1960s (Kemp et al., 2016); the flow of suspended sediment to coastal wetlands has declined from a mean of 390 Mt y^{-1} in the 1950s, to about 100 Mt y^{-1} since 1970 (Day et al., 2021).<br>Reduced bed loads that are hard to measure are another significant contributor to reduced sediment loads to coastal wetlands (Meade and Moody, 2010).
	Construction of flow barriers.	*Dams and impoundments on the mainstem Mississippi and Missouri Rivers and on the Illinois River in the Upper Mississippi River Basin.*<br>In the lower 110 km. of the Illinois river only 20% of the. original floodplain remains free of levees and seven locks and dams for navigation maintain a 2.74-m water depth within each pool (Lian et al., 2012).<br>A study comparing free-flowing and impounded habitats on a 171 km reach found that three indices of biotic health (index of biotic integrity, macroinvertebrate condition index and qualitative habitat evaluation index) were higher (healthier) in the free-flowing reaches than in the impounded sites (degraded) (Santucci et al., 2005).<br>Over the past century, more than 50,000 dams were built in the Mississippi River Basin (Xu et al., 2019).<br>Suspended sediment discharge from the Mississippi River has decreased by at least 50% since the 1950's due to impoundments and other engineering activities (Xu et al., 2019).

Driver category.	Key driver.	Examples within MARB.
2. River engineering.	Interbasin transfers (Lower Basin).	*Proposals to transfer water from the MARB to states in the Western U.S.*<br>Researchers from the International Water Resources Association suggested that the only sustainable path to water security for the states of California, Colorado and Nevada would be an interbasin transfer from the Mississippi River to the Colorado River (Natarajan et al., 2017). The infeasibility of this idea is documented in many places, see for example (Chawaga, 2022).<br>Ten states along the main stem of the Mississippi River are exploring a compact that would strengthen their collective power to keep water in their basin (Phillis and Salter, 2023).
3. Climate change	Increased mean annual near-surface air temperature.	*Temperatures have increased 0.8 °C since 1850.*<br>Temperatures trends have increased by 0.8 °C above historic levels since 1850 (Andresen et al., 2012).<br>There has been a warming trend of 0.029-0.037 °C/year for 1992-2013 in Illinois (Yeh and Wu, 2018).<br>The Gulf of Mexico is predicted to become a fully tropical sea by the end of the century, powering extreme weather events as a stronger heat reservoir (Day et al., 2021).
	Intensification of hydrologic cycle (changes in magnitudes, duration and frequency of precipitation/flow events).	*Both the frequency and intensity of rainfall and droughts are increasing.*<br>Data from the Gravity Recovery and Climate Experiment confirms that both the frequency and intensity of rainfall and droughts are increasing due to anthropogenic greenhouse gas emissions (Rohde, 2023).<br>More intense precipitation events move water downstream faster than historical events, resulting in more occurrences of river flooding and a higher probability of flooding events caused by both river flooding and marine storm surge (Dykstra and Dzwonkowski, 2021).<br>The 2012, 2017, and 2021 droughts are considered to be “flash droughts,” new events that develop much more quickly than droughts of the past and seem to be increasing in frequency and intensity (Otkin et al., 2022).<br>In the central United States, greenhouse gases are driving the increasing spring precipitation trends, which will lead to more frequent and severe flooding (Zhang and Villarini, 2021).
	Relative sea level rise (RSLR)	*Present-day RSLR rates in coastal Louisiana are among the highest in the world at ~12 mm per year.*<br>Modeling suggests, 25-40 years in the future, that the largest contributor to saline marsh loss will be coastal inundation (Reed et al., 2020).<br>Present-day RSLR rates in Louisiana are among the highest in the world at 12±8 mm per year. The Chenier Plain (SW Louisiana) may be more vulnerable to RSLR than the Mississippi River Delta with only 42% of wetlands keeping

		pace with RSLR vs. 65% in the Delta (Jankowski et al., 2017).<br>There is conflicting evidence regarding the resilience of coastal wetlands in Louisiana to RSLR and it may be better resolved by the time frame under consideration. Research suggests that wetland resilience to RSLR may be non-linear and after several decades the wetland system could diminish rapidly (Törnqvist et al., 2021).

Table S2. Potential management actions to improve ecosystem health in the Mississippi-Atchafalaya River Basin (MARB).

Management goal.	***Examples of management actions.***
**Reduce stressors**	
Minimize effects of land use change.	Minimize loss of existing forests, grasslands and wetlands by:<br>• providing conservation easements and other incentives to retain existing forests, grasslands and wetlands;<br>• requiring in-kind mitigation for loss of forests, grasslands and wetlands.
	Use voluntary or regulatory approaches to reduce unavoidable effects of land use change by:<br>• minimizing inputs of nutrients (e.g. via fertilizers and from concentrated animal feeding operations) and toxins through use of appropriate practices e.g., precision agriculture, agro-ecology practices, etc.;<br>• trapping and treating nutrients in agricultural and urban drainage through use of appropriate practices e.g., filter strips, wetlands;<br>• minimizing erosion on agricultural and urban lands through use of appropriate practices e.g., mulching, continuous living cover, grass waterways, grade control structures;<br>• trapping eroded sediment via silt fences, check dams, water and sediment control basins, etc.;<br>• minimizing changes in runoff through use of Low-Impact Development techniques and stormwater retention ponds in urban areas;<br>• minimizing water quality and quantity effects of expanded/intensified agricultural drainage (tile drainage and ditches) with ponds and wetlands at farm/small watershed scale;<br>• provide incentives for stream restoration;<br>• minimize groundwater withdrawals through improvements in irrigation efficiency and/or regulatory limits;<br>• limit inter-basin water transfers.
Minimize effects of river engineering.	Provide incentives for river restoration.
	Modify (e.g., notch) dams and levees, and re-operate dams to restore more natural flow regimes.
Mitigate climate change.	Minimize loss of existing carbon stocks by:<br>• providing conservation easements and other incentives for landowners to maintain existing carbon stocks in forests, grasslands and wetlands;<br>• requiring in-kind mitigation for loss of forests, grasslands and wetlands;<br>• promoting longer timber rotations.

Management goal.	***Examples of management actions.***
Mitigate climate change.	Use voluntary or regulatory approaches to reduce emissions of greenhouse gases by:<br>• reducing use of fossil fuel-based energy;<br>• improving management of nitrogen fertilizers;<br>• improving manure management;<br>improving feeding strategies for livestock.
	Promote the sequestration of carbon in perennial vegetation by:<br>• increasing carbon stocks in managed forests through selective thinning (to promote tree growth), regeneration after harvest and increasing structural complexity;<br>• enhancing carbon stocks in existing grasslands through proper grazing management, prescribed fire and planting of deep-rooted native perennial species;<br>• enhancing carbon stocks in existing wetlands by restoring the original hydrologic regime to keep soils wet;<br>• integrating perennial vegetation into agricultural landscapes through agroforestry, hedgerows, shelter belts, filter strips and riparian buffers;<br>• restoring grasslands restoration on marginal cropland e.g., through the Conservation Reserve Program;<br>• restoring wetlands (including floodplain forests) on marginal agricultural land.
**Restore and enhance ecosystem functions and processes**	
Improve nutrient cycling.	Support improved nutrient management through whole-farm and multi-year nutrient budgeting; promote practices that increase soil organic matter and soil microbial communities, controlled drainage, tailwater recycling on irrigated fields, and integration of crop and livestock production.
Improve nutrient retention and removal.	Promote restoration of nutrient sinks, e.g., ponds, floodplain and non-floodplain wetlands, and restored drainage ditches and streams.
Restore sediment retention in uplands.	Promote restoration of ponds and non-floodplain wetlands.
Restore sediment flow regime in river channels.	Support reconnection of river channels and floodplains (e.g., oxbow lake restoration, floodplain restoration, sediment diversions); create sediment bypass tunnels on dams (Lower Missouri River tributaries).
Increase water storage in landscape.	Promote water-harvesting techniques (e.g., bunds, ponds, percolation tanks) on farmland; restore wetlands (floodplain and non-floodplain).
Increase groundwater recharge.	Provide incentives for grassland and forest restoration, and for the development of infiltration basins.
Restore flow variability in streams and rivers	Re-operate dams for flow variability, especially flood pulses; reconfigure/setback levees; explore opportunities to restore a more natural flow regime by e.g., acquiring additional water rights.

Restore thermal buffering for streams and rivers	Encourage appropriate riparian planting; restore hyporheic connections to groundwater via stream restoration (e.g. use of pools and riffles, log dams).
Management goal.	***Examples of management actions.***
**Increase general resilience**	
Increase biodiversity at all scales.	Support the following: crop diversification (e.g., extended rotations and more diverse cropping systems); re-integration of crop and livestock production; landscape diversification (e.g., creation and/or restoration of hedgerows, prairie strips, woodlots, wetlands, and floodplain forests (including the use of diverse vegetation types in all of these); management of invasive species.
Increase terrestrial habitat connectivity to facilitate species dispersal.	Prioritize conservation funding to connecting existing habitat patches.
Increase aquatic habitat connectivity to facilitate species dispersal.	Identify sites suitable for the removal of artificial barriers or create passage structures through or around barriers; manage flow regimes for increased connectivity.
Create thermal refugia (aquatic).	Support the creation of riparian buffers; support stream restoration that enhances hyporheic exchange.

Table S3. Potential indicators to track ecosystem health.

[%, percentage; MARB, Mississippi-Atchafalaya River Basin; N, nitrogen; P, phosphorus; NDVI, normalized difference vegetation index; USGS, U.S. Geological Survey; CO2, carbon dioxide; CH4, methane; N2O, nitrous oxide]

Management goal.	***Potential indicators.***	***Potential data sources and/or approaches.***
**Reduce stressors.**		
Minimize effects of land use change.	Changes in % of Basin area in forest, grassland and wetland.	Remote sensing of land cover available through e.g. National Land Cover Database (USGS) https://www.mrlc.gov/data.<br>Land cover can be converted to land use using the schema developed in USGS' Wall-to-Wall Land Use Trends report, http://dx.doi.org/10.3133/ds948.
	For nutrients: changes in anthropogenic N and P inputs at county scale.	Inventory data for nutrients: anthropogenic N and P input data available in https://doi.org/10.5066/P9ZM964O.
	For toxins (e.g. pesticides): changes in amounts of pesticides applied.	Inventory data for pesticides: county-level data available from USGS at 5-year intervals; https://water.usgs.gov/nawqa/pnsp/usage/maps/.
	Changes in extent of winter vegetative cover (either NDVI or fractional green vegetation cover) and crop residue.	Multiple studies for cover crops for Upper Basin (e.g., Zhou *et al.*, 2022), but no routinely updated database at the scale of the MARB.
	Changes in length of tile drainage.	Nascent approaches use machine learning analysis of remotely sensed data; not yet suitable for routine deployment (Cho et al., 2019).
	Changes in channel sinuosity (e.g. due to ditching of headwater streams).	Remote sensing approaches are available at various scales. See discussion of mapping changes in channel sinuosity in (Boothroyd et al., 2021).
	Deviations from natural flow conditions (assessed using streamflow data from USGS gauge stations compared to estimated natural flow conditions).	Current streamflow data available at https://waterdata.usgs.gov/nwis/sw.<br>Data on estimated monthly natural flows described by (Miller et al., 2018) and available at: https://www.usgs.gov/data/natural-monthly-flow-estimates-conterminous-united-states-1950-2015.

Management goal.	***Potential indicators.***	***Potential data sources and/or approaches.***
Minimize effects of land use change.	Changes in volume of groundwater withdrawals.	USGS provides data on groundwater withdrawals by state and county at 5-year intervals at https://waterdata.usgs.gov/nwis (most recent data is for 2015). There are preliminary efforts to remotely sense groundwater withdrawals at greater spatial and temporal resolution, but so far these efforts have limited accuracy (Filippelli et al., 2022; Majumdar et al., 2021).
	Changes in volume of inter-basin water transfers	Most recent inventory of inter-basin transfers is available in Siddik et al. (2023).
Minimize effects of river engineering	Changes in area of aquatic habitat.	Satellite remote sensing approaches at large scales (Hugue et al., 2016; Torgersen et al., 2022).
	Changes in area of floodplain disconnected by levees.	Research is not specific to changes in floodplain area disconnected by levees. Flood inundation can be mapped using multi-spectral data, including changes in NDVI (Notti et al., 2018; Powell et al., 2014) and from data on water levels (altimetry, (Park, 2020) and radar backscatter (Notti et al., 2018)). Changes in flood inundation could be combined with changes in levee extent, mapped by changes in topography, land cover and distance from stream (Knox et al., 2022).
	Changes in number of stream and river flow barriers.	The Army Corps' National Inventory of Dams (https://nid.sec.usace.army.mil/#/) records dams meeting certain criteria (typically larger dams). Preliminary work has been done on mapping small (<1.5 m.) barriers using remote sensing (Buchanan et al., 2022).
Mitigate climate change	Changes in % of Basin area in forest, grassland and wetland.	Remote sensing of land cover available through e.g. National Land Cover Database (USGS) https://www.usgs.gov/centers/eros/science/national-land-cover-database. Land cover can be converted to land use using the schema developed in USGS' Wall-to-Wall Land Use Trends report, https://pubs.usgs.gov/ds/0948/ds948.pdf
	Changes in regional atmospheric concentrations of CO_2, CH_4 and N_2O.	Measurement of atmospheric concentrations of greenhouse gases using "top-down" (airborne, flux tower) measurements of greenhouse has concentrations coupled with inverse modeling. This approach has been demonstrated to detect real-time emissions of CH_4 from large regional sources and is being tested for N_2O (Joint Research Centre (European Commission) et al., 2018).
	Changes in amount of carbon uptake in tree/shrub biomass.	Remote sensing of forest structure coupled with carbon uptake (allometric) models. Data available at: GEDI Gridded Aboveground Biomass Density, https://doi.org/10.3334/ORNLDAAC/2299.

Management goal.	*Potential indicators.*	*Potential data sources and/or approaches.*
**Restore and enhance ecosystem functions and processes.**		
Improve nutrient cycling.	Changes in losses of N and P to air and water, quantified by changes in N and P balance.	Anthropogenic N and P balance data available from (Swaney et al., 2018; Swaney and Howarth, 2019).
Improve nutrient retention and removal.	Changes in area of hydrologically connected wetlands.<br>Changes in area of hydrologically connected floodplains.	Will require frequently updated wetland inundation maps. Preliminary remote sensing approach to mapping wetland connectivity in development (Qiusheng Wu et al., 2019).
Restore sediment retention and sinks.	Changes in area of hydrologically connected floodplains.	Flood inundation can be mapped using multi-spectral data, including changes in NDVI (Notti et al., 2018; Powell et al., 2014) and from data on water levels (altimetry, (Park, 2020) and radar backscatter (Notti et al., 2018)).
	Changes in annual volume of sediment accreted in and lost from the Delta.	Sediment surplus or deficit can be monitored (through the Coast-wide Reference Monitoring System in Louisiana, which tracks vertical accretion rates and sediment bulk density (Sanks et al., 2020)) and/or modelled (e.g. Allison *et al.*, 2012; Edmonds *et al.*, 2023).
Increase water storage in landscape.	Changes in water storage capacity in ponds and depressional wetlands; changes in potential water storage in reconnected floodplains.	Approaches for quantifying landscape-scale water storage described in (Green et al., 2019; Hou et al., 2022; Jones et al., 2018; Papa and Frappart, 2021).
Increase groundwater recharge.		Approaches for remotely quantifying regional groundwater recharge described in Wu et al., (2019b).
Increase flow variability in streams and rivers; restore functional flows.	Changes in multiple aspects of streamflow, e.g., floodplain inundation frequency, frequency and magnitude of peak flows during specific seasons; stability of base flows.	The challenge here is determining ecologically meaningful flow targets (see discussions in Bestgen et al., (2020) and Yarnell & Thoms, (2022).<br>One approach would be to set targets related to the natural flow regime: data described by (Miller et al., 2018) and available at: https://www.usgs.gov/data/natural-monthly-flow-estimates-conterminous-united-states-1950-2015<br>Current streamflow data available at https://waterdata.usgs.gov/nwis/sw.

Management goal.	*Potential indicators.*	*Potential data sources and/or approaches.*
Restore thermal buffering for streams and rivers	Changes in extent of riparian vegetation; changes in hyporheic discharge.	Satellite-based remote sensing of riparian vegetation remains challenging at regional-scale due to the low spatial resolution of much satellite data. Recent developments using LIDAR and WorldView-2 data (Michez et al., 2017; Rivas-Fandiño et al., 2022) offer promise but are not yet operational at scale. Thermal imaging can be used to map hyporheic zones (Dole-Olivier et al., 2019; Fullerton et al., 2017).
**Increase resilience.**		
Increase biodiversity at all scales.	Changes in various vegetative diversity indices.	Remote sensing of spectral variability (e.g. indices based on the coefficient of variation of NDVI) correlates with alpha (site-scale) and beta (cross-site) floral diversity (assumed to correlate with faunal diversity) (Wang and Gamon, 2019; Liccari et al., 2022).
Increase terrestrial habitat connectivity to facilitate species dispersal.	Changes in connectivity index at multiple scales.	Furberg et al. (2020) describe the use of high spatial resolution remotely sensed data to track changes in habitat connectivity.
Increase aquatic habitat connectivity to facilitate species dispersal.	Changes in hydrologic connectivity index at multiple scales.	A regional-scale example of using remote sensing to map changes in hydrologic connectivity over time is described in Xia et al. (2021).
Increase % watershed in perennial vegetation.	Changes in % perennial cover.	Remote sensing of land cover available through e.g. National Land Cover Database (USGS) https://www.usgs.gov/centers/eros/science/national-land-cover-database

Appendix A – Workshop summary

Workshop participants

Ed Arnett, *Chief Scientist, Theodore Roosevelt Conservation Partnership*
Kristen Bouska, *Ecologist, USGS Upper Midwest Environmental Sciences Center*
Daren Carlisle, *Ecological Studies Coordinator, USGS Earth Systems Processes Division*
Nathan De Jager, *Research Ecologist, USGS Upper Midwest Environmental Sciences Center*
Joe Flotemersch, *Research Biologist, EPA National Exposure Research Laboratory*
Jeff Frey, *Deputy Director, USGS Ohio-Kentucky-Indiana Water Science Center*
Madison Goff, *Workshop Facilitator*
Heather Golden, *Research Physical Scientist, EPA National Center for Measurement and Modeling*
Ryan Hill, *Geospatial Aquatic Ecologist, EPA National Health and Environmental Effects Research Laboratory*
Tara Hohman, *Conservation Science Associate, UMR Program, National Audubon Society*
Jeff Houser, *Research Ecologist, USGS Upper Midwest Environmental Sciences Center*
Erik Johnson, *Conservation Director for Audubon Delta, National Audubon Society*
Shamitha Keerthi, *Agriculture and Water Quality Scientist, North America, The Nature Conservancy*
Richard Keim, *Professor, Louisiana State University, School of Renewable Natural Resources*
Sammy King, *USGS Leader, USGS Louisiana Cooperative Fish & Wildlife Research Unit; Adjunct Associate Professor, Louisiana State University School of Renewable Natural Resources*
Barb Kleiss, *Research Professor, River and Science Engineering, Tulane University*
Tyler Lark, *Assistant Scientist, Center for Sustainability and the Global Environment, University of Wisconsin*
Kim Lutz, *Executive Director, America's Watershed Initiative*
Eileen McLellan, *Lead Senior Scientist, Environmental Defense Fund*
Bryan Piazza, *Science Lead for Mississippi Basin, The Nature Conservancy*
Alisha Renfro, *Coastal Scientist, Mississippi River Delta Restoration Program, National Wildlife Federation*
Dale Robertson, *Research Hydrologist, USGS Upper Midwest Water Science Center*
Keith Schilling, *State Geologist of Iowa; Director of the Iowa Geological Survey at the University of Iowa.*
Travis Schmidt, *Research Ecologist, Wyoming-Montana Water Science Center*
Shelby Shelton, *Manager, Climate-Smart Agriculture, Environmental Defense Fund*
Kelly Suttles, *Senior Research Analyst, Environmental Defense Fund*
Ian Waite, *Research Biologist, Oregon Water Science Center, United States Geological Survey*
Kirsten Wallace, *Executive Director, Upper Mississippi River Basin Association*

Workshop Process and Outcomes

During the workshop, participants began by highlighting the ecosystem components (e.g., forests, floodplains) most important to them or their organization. This exercise allowed the participants to see the diversity of interests across the Mississippi River ecosystem, to discuss potential high-level commonalities, and to begin to identify potential targets. Then, the participants agreed upon stressors and important ecological functions in the Mississippi River ecosystem, which was refined post-workshop into a conceptual model of opportunities for improving ecosystem health. Participants also identified potential indicators to measure progress towards a healthy Mississippi River ecosystem. This list of indicators was then refined by a small group of external scientists in a follow-up discussion. Finally, a small group of NGO partners reviewed and organized the results into a vision statement with narrative goals and associated targets. By utilizing ecosystem-wide thinking throughout the process, the participants were able to think beyond their section of riverscape or landscape.

The workshop and its two follow-up small group discussions generated the following consensus outcomes:

- A vision and goals for a healthy Mississippi River ecosystem;
- Identification of those aspects of ecosystem condition (water quality, hydrologic alteration, and habitat) for which numeric targets would need to be developed;
- A conceptual model describing opportunities to improve ecosystem health by reducing stressors, improving function, and increasing resilience;
- A suite of potential indicators to measure progress in improving ecosystem health;
- A proposal for using indicators in an adaptive management framework to guide overall ecosystem restoration as science evolves.

References

Abrams, R., Markus, M., 2021. Does the Doctrine of Equitable Apportionment Apply to Conflicts between States over Groundwater Resources When Such Resources Are Derived from an Aquifer That Lies Beneath More than One State? 49 PREVIEW U.S. SUP. CT. CAS. 3.

Allison, M.A., Demas, C.R., Ebersole, B.A., Kleiss, B.A., Little, C.D., Meselhe, E.A., Powell, N.J., Pratt, T.C., Vosburg, B.M., 2012. A water and sediment budget for the lower Mississippi–Atchafalaya River in flood years 2008–2010: Implications for sediment discharge to the oceans and coastal restoration in Louisiana. Journal of Hydrology 432–433, 84–97. https://doi.org/10.1016/j.jhydrol.2012.02.020

Andresen, J., Hilberg, S., Kunkel, K., 2012. Historical Climate and Climate Trends in the Midwestern USA. Great Lakes Integrated Sciences and Assessments Center.

Bajgain, R., Xiao, X., Basara, J., Wagle, P., Zhou, Y., Mahan, H., Gowda, P., McCarthy, H.R., Northup, B., Neel, J., Steiner, J., 2018. Carbon dioxide and water vapor fluxes in winter wheat and tallgrass prairie in central Oklahoma. Science of The Total Environment 644, 1511–1524. https://doi.org/10.1016/j.scitotenv.2018.07.010

Becker, J.P., Peterson, E.W., 2022. Stream Recovery Post Channelization: A Case Study of Low-Gradient Streams in Central Illinois, USA. Hydrology 9, 160. https://doi.org/10.3390/hydrology9090160

Berkowitz, J.F., Johnson, D.R., Price, J.J., 2020. Forested Wetland Hydrology in a Large Mississippi River Tributary System. Wetlands 40, 1133–1148. https://doi.org/10.1007/s13157-019-01249-5

Bestgen, K.R., Poff, N.L., Baker, D.W., Bledsoe, B.P., Merritt, D.M., Lorie, M., Auble, G.T., Sanderson, J.S., Kondratieff, B.C., 2020. Designing flows to enhance ecosystem functioning in heavily altered rivers. Ecological Applications 30, e02005. https://doi.org/10.1002/eap.2005

Boland-Brien, S.J., Basu, N.B., Schilling, K.E., 2014. Homogenization of spatial patterns of hydrologic response in artificially drained agricultural catchments: HOMOGENIZATION OF HYDROLOGIC RESPONSE IN AGRICULTURAL CATCHMENTS. Hydrol. Process. 28, 5010–5020. https://doi.org/10.1002/hyp.9967

Boothroyd, R.J., Williams, R.D., Hoey, T.B., Barrett, B., Prasojo, O.A., 2021. Applications of Google Earth Engine in fluvial geomorphology for detecting river channel change. WIREs Water 8, e21496. https://doi.org/10.1002/wat2.1496

Borchert, S.M., Osland, M.J., Enwright, N.M., Griffith, K.T., 2018. Coastal wetland adaptation to sea level rise: Quantifying potential for landward migration and coastal squeeze. Journal of Applied Ecology 55, 2876–2887. https://doi.org/10.1111/1365-2664.13169

Buchanan, B.P., Sethi, S.A., Cuppett, S., Lung, M., Jackman, G., Zarri, L., Duvall, E., Dietrich, J., Sullivan, P., Dominitz, A., Archibald, J.A., Flecker, A., Rahm, B.G., 2022. A machine learning approach to identify barriers in stream networks demonstrates high prevalence of unmapped riverine dams. Journal of Environmental Management 302, 113952. https://doi.org/10.1016/j.jenvman.2021.113952

Carstens, D., Amer, R., 2019. Spatio-temporal analysis of urban changes and surface water quality. Journal of Hydrology 569, 720–734. https://doi.org/10.1016/j.jhydrol.2018.12.033

Chawaga, P., 2022. Can A Pipeline Really Bring Drinking Water From Mississippi To The West? [WWW Document]. URL https://www.wateronline.com/doc/can-a-pipeline-really-bring-drinking-water-from-mississippi-to-the-west-0001 (accessed 11.7.22).

Cho, E., Jacobs, J.M., Jia, X., Kraatz, S., 2019. Identifying Subsurface Drainage using Satellite Big Data and Machine Learning via Google Earth Engine. Water Resources Research 55, 8028–8045. https://doi.org/10.1029/2019WR024892

Day, J.W., Hunter, R., Kemp, G.P., Moerschbaecher, M., Brantley, C.G., 2021. The “Problem” of New Orleans and Diminishing Sustainability of Mississippi River Management—Future Options. Water 13, 813. https://doi.org/10.3390/w13060813

Dole-Olivier, M.-J., Wawzyniak, V., Creuzé des Châtelliers, M., Marmonier, P., 2019. Do thermal infrared (TIR) remote sensing and direct hyporheic measurements (DHM) similarly detect river-groundwater exchanges? Study along a 40 km-section of the Ain River (France). Science of The Total Environment 646, 1097–1110. https://doi.org/10.1016/j.scitotenv.2018.07.294

Dykstra, S.L., Dzwonkowski, B., 2021. The Role of Intensifying Precipitation on Coastal River Flooding and Compound River-Storm Surge Events, Northeast Gulf of Mexico. Water Resources Research 57, e2020WR029363. https://doi.org/10.1029/2020WR029363

Edmonds, D.A., Toby, S.C., Siverd, C.G., Twilley, R., Bentley, S.J., Hagen, S., Xu, K., 2023. Land loss due to human-altered sediment budget in the Mississippi River Delta. Nat Sustain. https://doi.org/10.1038/s41893-023-01081-0

Elliott, S.M., VanderMeulen, D.D., 2017. A regional assessment of chemicals of concern in surface waters of four Midwestern United States national parks. Science of The Total Environment 579, 1726–1735. https://doi.org/10.1016/j.scitotenv.2016.11.114

Fernandez-Cornejo, J., Nehring, R.F., Osteen, C., Wechsler, S., Martin, A., Vialou, A., 2014. Pesticide Use in U.S. Agriculture: 21 Selected Crops, 1960-2008. https://doi.org/10.2139/ssrn.2502986

Filippelli, S.K., Sloggy, M.R., Vogeler, J.C., Manning, D.T., Goemans, C., Senay, G.B., 2022. Remote sensing of field-scale irrigation withdrawals in the central Ogallala aquifer region. Agricultural Water Management 271, 107764. https://doi.org/10.1016/j.agwat.2022.107764

Fullerton, A.H., Torgersen, C.E., Lawler, J.J., Steel, E.A., Ebersole, J.L., Lee, S.Y., 2017. Longitudinal thermal heterogeneity in rivers and refugia for coldwater species: effects of scale and climate change. Aquat Sci 80, 3. https://doi.org/10.1007/s00027-017-0557-9

Furberg, D., Ban, Y., Mörtberg, U., 2020. Monitoring Urban Green Infrastructure Changes and Impact on Habitat Connectivity Using High-Resolution Satellite Data. Remote Sensing 12, 3072. https://doi.org/10.3390/rs12183072

Gad, A.K., Toner, K., Benfield, M.C., Midway, S.R., 2023. Microplastics in mainstem Mississippi River fishes. Frontiers in Environmental Science 10.

Golden, H.E., Lane, C.R., Rajib, A., Wu, Q., 2021. Improving global flood and drought predictions: integrating non-floodplain wetlands into watershed hydrologic models. Environ. Res. Lett. 16, 091002. https://doi.org/10.1088/1748-9326/ac1fbc

Green, D.I.S., McDeid, S.M., Crumpton, W.G., 2019. Runoff Storage Potential of Drained Upland Depressions on the Des Moines Lobe of Iowa. JAWRA Journal of the American Water Resources Association 55, 543–558. https://doi.org/10.1111/1752-1688.12738

Hanberry, B.B., Abrams, M.D., 2018. Recognizing loss of open forest ecosystems by tree densification and land use intensification in the Midwestern USA. Reg Environ Change 18, 1731–1740. https://doi.org/10.1007/s10113-018-1299-5

Hou, J., Van Dijk, A.I.J.M., Renzullo, L.J., 2022. Merging Landsat and airborne LiDAR observations for continuous monitoring of floodplain water extent, depth and volume. Journal of Hydrology 609, 127684. https://doi.org/10.1016/j.jhydrol.2022.127684

Hu, S., Shrestha, P., 2020. Examine the impact of land use and land cover changes on peak discharges of a watershed in the midwestern United States using the HEC-HMS model.

Papers in Applied Geography 6, 101–118. https://doi.org/10.1080/23754931.2020.1732447
Hugue, F., Lapointe, M., Eaton, B.C., Lepoutre, A., 2016. Satellite-based remote sensing of running water habitats at large riverscape scales: Tools to analyze habitat heterogeneity for river ecosystem management. Geomorphology 253, 353–369. https://doi.org/10.1016/j.geomorph.2015.10.025
Jankowski, K.L., Törnqvist, T.E., Fernandes, A.M., 2017. Vulnerability of Louisiana's coastal wetlands to present-day rates of relative sea-level rise. Nat Commun 8, 14792. https://doi.org/10.1038/ncomms14792
Jaynes, D.B., James, D.E., n.d. The Extent of Farm Drainage in the United States.
Jiang, Y., Fu, P., Weng, Q., 2015. Assessing the Impacts of Urbanization-Associated Land Use/Cover Change on Land Surface Temperature and Surface Moisture: A Case Study in the Midwestern United States. Remote Sensing 7, 4880–4898. https://doi.org/10.3390/rs70404880
Joint Research Centre (European Commission), Houweling, S., Chevalier, F., Strogies, M., Dlugokencky, E., Ramonet, M., Pandey, S., Hammer, S., Röckmann, T., Weiss, R.F., Reimann, S., Sussams, J., Aardenne, J. van, Bovensmann, H., Maione, M., Thompson, R.L., Levin, I., Montzka, S., Danila, A., Crisp, D., Kort, E.A., Schmidt, M., Engelen, R., Gerbig, C., Peters, W., Henne, S., Bergamaschi, P., Miller, J., Basu, S., Peylin, P., Ciais, P., Pinty, B., Janssens-Maenhout, G., Manning, A.J., Günther, D., Vogel, F., Karstens, U., Brunner, D., Tarasova, O., Vermeulen, T., Meijer, Y., 2018. Atmospheric monitoring and inverse modelling for verification of greenhouse gas inventories. Publications Office of the European Union, LU.
Jones, C.N., Evenson, G.R., McLaughlin, D.L., Vanderhoof, M.K., Lang, M.W., McCarty, G.W., Golden, H.E., Lane, C.R., Alexander, L.C., 2018. Estimating restorable wetland water storage at landscape scales. Hydrological Processes 32, 305–313. https://doi.org/10.1002/hyp.11405
Knox, R.L., Morrison, R.R., Wohl, E.E., 2022. Identification of Artificial Levees in the Contiguous United States. Water Resources Research 58, e2021WR031308. https://doi.org/10.1029/2021WR031308
Lark, T.J., Spawn, S.A., Bougie, M., Gibbs, H.K., 2020. Cropland expansion in the United States produces marginal yields at high costs to wildlife. Nat Commun 11, 4295. https://doi.org/10.1038/s41467-020-18045-z
Lian, Y., You, J.-Y., Sparks, R., Demissie, M., 2012. Impact of Human Activities to Hydrologic Alterations on the Illinois River. J. Hydrol. Eng. 17, 537–546. https://doi.org/10.1061/(ASCE)HE.1943-5584.0000465
Liccari, F., Sigura, M., Bacaro, G., 2022. Use of Remote Sensing Techniques to Estimate Plant Diversity within Ecological Networks: A Worked Example. Remote Sensing 14, 4933. https://doi.org/10.3390/rs14194933
Majumdar, S., Smith, R., Conway, B.D., Butler, J.J., Lakshmi, V., Dagli, C.H., 2021. Estimating Local-Scale Groundwater Withdrawals Using Integrated Remote Sensing Products and Deep Learning, in: 2021 IEEE International Geoscience and Remote Sensing Symposium IGARSS. Presented at the 2021 IEEE International Geoscience and Remote Sensing Symposium IGARSS, pp. 4304–4307. https://doi.org/10.1109/IGARSS47720.2021.9554784
Meade, R.H., Moody, J.A., 2010. Causes for the decline of suspended-sediment discharge in the Mississippi River system, 1940–2007. Hydrological Processes 24, 35–49. https://doi.org/10.1002/hyp.7477
Michez, A., Piégay, H., Lejeune, P., Claessens, H., 2017. Multi-temporal monitoring of a regional riparian buffer network (>12,000 km) with LiDAR and photogrammetric point

clouds. Journal of Environmental Management, Piégay & Lamouroux “Enlarging spatial and temporal scales for biophysical diagnosis and sustainable river management” 202, 424–436. https://doi.org/10.1016/j.jenvman.2017.02.034
Miller, M.P., Carlisle, D.M., Wolock, D.M., Wieczorek, M., 2018. A Database of Natural Monthly Streamflow Estimates from 1950 to 2015 for the Conterminous United States. JAWRA Journal of the American Water Resources Association 54, 1258–1269. https://doi.org/10.1111/1752-1688.12685
Mitsch, W.J., Day, J.W., 2006. Restoration of wetlands in the Mississippi–Ohio–Missouri (MOM) River Basin: Experience and needed research. Ecological Engineering 26, 55–69. https://doi.org/10.1016/j.ecoleng.2005.09.005
Morefield, P.E., LeDuc, S.D., Clark, C.M., Iovanna, R., 2016. Grasslands, wetlands, and agriculture: the fate of land expiring from the Conservation Reserve Program in the Midwestern United States. Environ. Res. Lett. 11, 094005. https://doi.org/10.1088/1748-9326/11/9/094005
Murray, A.S., Biedenharn, D.S., 2022. Sediment supply from bank caving on the Lower Mississippi River, 1765 to present (Report). United States. Army. Corps of Engineers. Mississippi Valley Division.
Natarajan, D.P.M., Ponnavaiko, D.M., Kalloikar, S., Rangaraju, D.G., Ganesh, S., 2017. Achieving Permanent Water Security to Colorado River Basin is a Dream, Without Transbasin Diversion from Mississippi River 15.
Nienhuis, J.H., Törnqvist, T.E., Jankowski, K.L., Fernandes, A.M., Keogh, M.E., 2017. A New Subsidence Map for Coastal Louisiana. GSAT 60–61. https://doi.org/10.1130/GSATG337GW.1
Notti, D., Giordan, D., Caló, F., Pepe, A., Zucca, F., Galve, J.P., 2018. Potential and Limitations of Open Satellite Data for Flood Mapping. Remote Sensing 10, 1673. https://doi.org/10.3390/rs10111673
Otkin, J.A., Woloszyn, M., Wang, H., Svoboda, M., Skumanich, M., Pulwarty, R., Lisonbee, J., Hoell, A., Hobbins, M., Haigh, T., Cravens, A.E., 2022. Getting ahead of Flash Drought: From Early Warning to Early Action. Bulletin of the American Meteorological Society 103, E2188–E2202. https://doi.org/10.1175/BAMS-D-21-0288.1
Papa, F., Frappart, F., 2021. Surface Water Storage in Rivers and Wetlands Derived from Satellite Observations: A Review of Current Advances and Future Opportunities for Hydrological Sciences. Remote Sensing 13, 4162. https://doi.org/10.3390/rs13204162
Park, E., 2020. Characterizing channel-floodplain connectivity using satellite altimetry: Mechanism, hydrogeomorphic control, and sediment budget. Remote Sensing of Environment 243, 111783. https://doi.org/10.1016/j.rse.2020.111783
Phillis, M., Salter, J., 2023. Dry states taking Mississippi River water isn’t a new idea. But some mayors want to kill it. AP News.
Powell, S.J., Jakeman, A., Croke, B., 2014. Can NDVI response indicate the effective flood extent in macrophyte dominated floodplain wetlands? Ecological Indicators 45, 486–493. https://doi.org/10.1016/j.ecolind.2014.05.009
Prestegaard, K.L., Matherne, A.M., Shane, B., Houghton, K., O’Connell, M., Katyl, N., 1994. Spatial variations in the magnitude of the 1993 floods, Raccoon River basin, Iowa, in: Morisawa, M. (Ed.), Geomorphology and Natural Hazards. Elsevier, Amsterdam, pp. 169–182. https://doi.org/10.1016/B978-0-444-82012-9.50016-5
Reba, M.L., Massey, J.H., 2020. Surface Irrigation in the Lower Mississippi River Basin: Trends and Innovations. Transactions of the ASABE 63, 1305–1314. https://doi.org/10.13031/trans.13970
Reba, M.L., Massey, J.H., Adviento-Borbe, M.A., Leslie, D., Yaeger, M.A., Anders, M., Farris, J., 2017. Aquifer Depletion in the Lower Mississippi River Basin: Challenges and Solutions.

Journal of Contemporary Water Research & Education 162, 128–139. https://doi.org/10.1111/j.1936-704X.2017.03264.x
Reed, D., Wang, Y., Meselhe, E., White, E., 2020. Modeling wetland transitions and loss in coastal Louisiana under scenarios of future relative sea-level rise. Geomorphology 352, 106991. https://doi.org/10.1016/j.geomorph.2019.106991
Remo, J.W.F., Khanal, A., Pinter, N., 2013. Assessment of chevron dikes for the enhancement of physical-aquatic habitat within the Middle Mississippi River, USA. Journal of Hydrology 501, 146–162. https://doi.org/10.1016/j.jhydrol.2013.07.007
Rivas-Fandiño, P., Acuña-Alonso, C., Novo, A., Pacheco, F.A.L., Álvarez, X., 2022. Assessment of high spatial resolution satellite imagery for monitoring riparian vegetation: riverine management in the smallholding. Environ Monit Assess 195, 81. https://doi.org/10.1007/s10661-022-10667-8
Rohde, M.M., 2023. Floods and droughts are intensifying globally. Nat Water 1, 226–227. https://doi.org/10.1038/s44221-023-00047-y
Sanks, K.M., Shaw, J.B., Naithani, K., 2020. Field-Based Estimate of the Sediment Deficit in Coastal Louisiana. Journal of Geophysical Research: Earth Surface 125, e2019JF005389. https://doi.org/10.1029/2019JF005389
Santucci, V.J., Gephard, S.R., Pescitelli, S.M., 2005. Effects of Multiple Low-Head Dams on Fish, Macroinvertebrates, Habitat, and Water Quality in the Fox River, Illinois. North American Journal of Fisheries Management 25, 975–992. https://doi.org/10.1577/M03-216.1
Schilling, K.E., Jones, C.S., Seeman, A., Bader, E., Filipiak, J., 2012. Nitrate-nitrogen patterns in engineered catchments in the upper Mississippi River basin. Ecological Engineering 42, 1–9. https://doi.org/10.1016/j.ecoleng.2012.01.026
Schramm, H., Ickes, B., 2013. The Mississippi River: A Place for Fish.
Siddik, Md.A.B., Dickson, K.E., Rising, J., Ruddell, B.L., Marston, L.T., 2023. Interbasin water transfers in the United States and Canada. Sci Data 10, 27. https://doi.org/10.1038/s41597-023-01935-4
Skees, A.J., Foppe, K.S., Loganathan, B., Subedi, B., 2018. Contamination profiles, mass loadings, and sewage epidemiology of neuropsychiatric and illicit drugs in wastewater and river waters from a community in the Midwestern United States. Science of The Total Environment 631–632, 1457–1464. https://doi.org/10.1016/j.scitotenv.2018.03.060
Stackpoole, S.M., Shoda, M.E., Medalie, L., Stone, W.W., 2021. Pesticides in US Rivers: Regional differences in use, occurrence, and environmental toxicity, 2013 to 2017. Science of The Total Environment 787, 147147. https://doi.org/10.1016/j.scitotenv.2021.147147
Swaney, D.P., Howarth, R.W., 2019. Phosphorus use efficiency and crop production: Patterns of regional variation in the United States, 1987–2012. Science of The Total Environment 685, 174–188. https://doi.org/10.1016/j.scitotenv.2019.05.228
Swaney, D.P., Howarth, R.W., Hong, B., 2018. County, subregional and regional nitrogen data derived from the Net Anthropogenic Nitrogen Inputs (NANI) toolbox. Data in Brief 18, 1877–1888. https://doi.org/10.1016/j.dib.2018.04.098
Torgersen, C.E., Le Pichon, C., Fullerton, A.H., Dugdale, S.J., Duda, J.J., Giovannini, F., Tales, É., Belliard, J., Branco, P., Bergeron, N.E., Roy, M.L., Tonolla, D., Lamouroux, N., Capra, H., Baxter, C.V., 2022. Riverscape approaches in practice: perspectives and applications. Biological Reviews 97, 481–504. https://doi.org/10.1111/brv.12810
Törnqvist, T.E., Cahoon, D.R., Morris, J.T., Day, J.W., 2021. Coastal Wetland Resilience, Accelerated Sea-Level Rise, and the Importance of Timescale. AGU Advances 2, e2020AV000334. https://doi.org/10.1029/2020AV000334

University of Illinois Extension, 2023. Drainage Tile History in the U.S. [WWW Document]. Bioreactors, Water Table Management, and Water Quality. URL https://web.extension.illinois.edu/bioreactors/history.cfm (accessed 6.12.23).
US EPA, O., 2022. Report on the Environment (ROE) Indicators – Fertilizer Applied for Agricultural Purposes [WWW Document]. Fertilizer Applied for Agricultural Purposes. URL https://cfpub.epa.gov/roe/indicator.cfm?i=55 (accessed 6.12.23).
USDA - National Agricultural Statistics Service, 2019. 2017 Census of Agriculture Volume 1, Chapter 1: U.S. National Level Data (No. AC-17-A-51). United States Department of Agriculture.
Valayamkunnath, P., Barlage, M., Chen, F., Gochis, D.J., Franz, K.J., 2020. Mapping of 30-meter resolution tile-drained croplands using a geospatial modeling approach. Sci Data 7, 257. https://doi.org/10.1038/s41597-020-00596-x
Wu, Qiusheng, Lane, C.R., Li, X., Zhao, K., Zhou, Y., Clinton, N., DeVries, B., Golden, H.E., Lang, M.W., 2019. Integrating LiDAR data and multi-temporal aerial imagery to map wetland inundation dynamics using Google Earth Engine. Remote Sensing of Environment 228, 1–13. https://doi.org/10.1016/j.rse.2019.04.015
Wu, Qifan, Si, B., He, H., Wu, P., 2019. Determining Regional-Scale Groundwater Recharge with GRACE and GLDAS. Remote Sensing 11, 154. https://doi.org/10.3390/rs11020154
Xia, Y., Fang, C., Lin, H., Li, H., Wu, B., 2021. Spatiotemporal Evolution of Wetland Eco-Hydrological Connectivity in the Poyang Lake Area Based on Long Time-Series Remote Sensing Images. Remote Sensing 13, 4812. https://doi.org/10.3390/rs13234812
Xu, K., Bentley, S.J., Day, J.W., Freeman, A.M., 2019. A review of sediment diversion in the Mississippi River Deltaic Plain. Estuarine, Coastal and Shelf Science 225, 106241. https://doi.org/10.1016/j.ecss.2019.05.023
Yarnell, S.M., Thoms, M., 2022. Enhancing the functionality of environmental flows through an understanding of biophysical processes in the riverine landscape. Frontiers in Environmental Science 10.
Yeh, P.J.-F., Wu, C., 2018. Recent Acceleration of the Terrestrial Hydrologic Cycle in the U.S. Midwest. Journal of Geophysical Research: Atmospheres 123, 2993–3008. https://doi.org/10.1002/2017JD027706
Zhang, W., Villarini, G., 2021. Greenhouse gases drove the increasing trends in spring precipitation across the central USA. Philosophical Transactions of the Royal Society A: Mathematical, Physical and Engineering Sciences 379, 20190553. https://doi.org/10.1098/rsta.2019.0553
Zhang, X., Lark, T.J., Clark, C.M., Yuan, Y., LeDuc, S.D., 2021. Grassland-to-cropland conversion increased soil, nutrient, and carbon losses in the US Midwest between 2008 and 2016. Environ. Res. Lett. 16, 054018. https://doi.org/10.1088/1748-9326/abecbe
Zhou, B., He, H.S., Nigh, T.A., Schulz, J.H., 2012. Mapping and analyzing change of impervious surface for two decades using multi-temporal Landsat imagery in Missouri. International Journal of Applied Earth Observation and Geoinformation 18, 195–206. https://doi.org/10.1016/j.jag.2012.02.003
Zhou, Q., Guan, K., Wang, Sheng, Jiang, C., Huang, Y., Peng, B., Chen, Z., Wang, Sibo, Hipple, J., Schaefer, D., Qin, Z., Stroebel, S., Coppess, J., Khanna, M., Cai, Y., 2022. Recent Rapid Increase of Cover Crop Adoption Across the U.S. Midwest Detected by Fusing Multi-Source Satellite Data. Geophysical Research Letters 49, e2022GL100249. https://doi.org/10.1029/2022GL100249
Zubair, O.A., Ji, W., Festus, O., 2019. Urban Expansion and the Loss of Prairie and Agricultural Lands: A Satellite Remote-Sensing-Based Analysis at a Sub-Watershed Scale. Sustainability 11, 4673. https://doi.org/10.3390/su11174673
